# Bioenergy production and sustainable development: science base for policymaking remains limited

**DOI:** 10.1111/gcbb.12338

**Published:** 2016-03-23

**Authors:** Carmenza Robledo‐Abad, Hans‐Jörg Althaus, Göran Berndes, Simon Bolwig, Esteve Corbera, Felix Creutzig, John Garcia‐Ulloa, Anna Geddes, Jay S. Gregg, Helmut Haberl, Susanne Hanger, Richard J. Harper, Carol Hunsberger, Rasmus K. Larsen, Christian Lauk, Stefan Leitner, Johan Lilliestam, Hermann Lotze‐Campen, Bart Muys, Maria Nordborg, Maria Ölund, Boris Orlowsky, Alexander Popp, Joana Portugal‐Pereira, Jürgen Reinhard, Lena Scheiffle, Pete Smith

**Affiliations:** ^1^ Department of Environmental Systems ScienceUSYS TdLabETH ZürichUniversitätstrasse 228092ZurichSwitzerland; ^2^ Helvetas Swiss IntercooperationMaulbeerstr. 10CH‐3001BernSwitzerland; ^3^ Foundation for Global Sustainability (ffgs)Reitergasse 118004ZürichSwitzerland; ^4^ Lifecycle Consulting AlthausBruechstr. 1328706MeilenSwitzerland; ^5^ Department of Energy and EnvironmentChalmers University of TechnologySE 41296GothenburgSweden; ^6^ DTU Management EngineeringTechnical University of Denmark4000RoskildeDenmark; ^7^ Institute of Environmental Science and Technology, and Department of Economics & Economic HistoryUniversitat Autònoma de Barcelona08193BarcelonaSpain; ^8^ Mercator Research Institute on Global Commons and Climate Change & Technical University Berlin10829BerlinGermany; ^9^ Institute of Terrestrial EcosystemsETH ZürichUniversitätstrasse 22, 8092ZurichSwitzerland; ^10^ Institute for Environmental DecisionsETH ZürichClimate Policy GroupUniversitätstrasse 228092ZurichSwitzerland; ^11^ Institute of Social Ecology Vienna (SEC)Alpen‐Adria Universitaet (AAU)Schottenfeldgasse 291070ViennaAustria; ^12^ International Institute for Applied Systems AnalysisSchlossplatz 1LaxenburgAustria; ^13^ School of Veterinary and Life SciencesMurdoch UniversitySouth StreetMurdochWA6150Australia; ^14^ Department of GeographyUniversity of Western OntarioLondonONN6A 5C2Canada; ^15^ Stockholm Environment Institute (SEI)Linnégatan 87D115 23 StockholmPostbox 24218104 51StockholmSweden; ^16^ Potsdam Institute for Climate Impact Research (PIK)PO Box 60120314412PotsdamGermany; ^17^ Humboldt‐University zu BerlinUnter den Linden 610099BerlinGermany; ^18^ Division of Forest, Nature and LandscapeUniversity of Leuven (KU Leuven)Celestijnenlaan 200E box 2411BE‐ 3001LeuvenBelgium; ^19^ Centre for Environment and Sustainability – GMVUniversity of GothenburgAschebergsgatan 44GöteborgSweden; ^20^ Climate‐Babel; ^21^ Energy Planning ProgramCOPPE, Federal University of Rio de JaneiroCentro de TecnologiaSala C‐211, C.P. 68565, Cidade UniversitáriaIlha do Fundão21941‐972Rio de JaneiroRJBrazil; ^22^ Informatics and Sustainability Research GroupSwiss Federal Institute for Material Testing and ResearchEmpa, Ueberlandstrasse 1298600DuebendorfSwitzerland; ^23^ Institute of Biological & Environmental SciencesClimateXChange and Scottish Food Security Alliance‐CropsUniversity of Aberdeen23 St Machar DriveAberdeenAB24 3UUUK

**Keywords:** agriculture, bioenergy, food security, forestry, mitigation, sustainable development

## Abstract

The possibility of using bioenergy as a climate change mitigation measure has sparked a discussion of whether and how bioenergy production contributes to sustainable development. We undertook a systematic review of the scientific literature to illuminate this relationship and found a limited scientific basis for policymaking. Our results indicate that knowledge on the sustainable development impacts of bioenergy production is concentrated in a few well‐studied countries, focuses on environmental and economic impacts, and mostly relates to dedicated agricultural biomass plantations. The scope and methodological approaches in studies differ widely and only a small share of the studies sufficiently reports on context and/or baseline conditions, which makes it difficult to get a general understanding of the attribution of impacts. Nevertheless, we identified regional patterns of positive or negative impacts for all categories – environmental, economic, institutional, social and technological. In general, economic and technological impacts were more frequently reported as positive, while social and environmental impacts were more frequently reported as negative (with the exception of impacts on direct substitution of GHG emission from fossil fuel). More focused and transparent research is needed to validate these patterns and develop a strong science underpinning for establishing policies and governance agreements that prevent/mitigate negative and promote positive impacts from bioenergy production.

## Introduction

During the last decades, developed and developing countries have introduced policies to encourage the use of bioenergy including *i.a*. the Brazilian National Alcohol Program (ProAlcool), the US Renewable Fuel Standard (RFS), the EU's Renewable Energy Directive (RED), the Alternative Energy Development Plan (AEDP) in Thailand, and the Indian National Policy on Biofuels (Sorda *et al*., 2010). The promotion of bioenergy as a climate change mitigation measure has sparked a intensive discussion concerning potential impacts on sustainable development. Commonly mentioned positive impacts focus on opportunities for new uses of land, economic growth, climate change mitigation, increased energy security and employment (Smeets *et al*., 2007; Nijsen *et al*., 2012; Mendes Souza *et al*., 2015). On the other hand, there are concerns about potential disruption to food security and rural livelihoods, direct and indirect greenhouse gas (GHG) emissions from land use change, enhanced water scarcity, ecological impacts, increased rural poverty, and displacement of small‐scale farmers, pastoralists and forest users (Dauvergne & Neville, 2010; Delucchi, 2010; German *et al*., 2011; Gamborg *et al*., 2014; Hejazi *et al*., 2015).

How bioenergy interacts with sustainable development has become a key scientific question as demand for bioenergy increases globally. The recent Intergovernmental Panel on Climate Change (IPCC) Working Group III contribution to the Fifth Assessment Report (WGIII AR5) highlights the relationship between context conditions, the use of bioenergy as a mitigation option and the impacts on sustainable development. Discussing impacts of bioenergy on sustainable development, the IPCC WGIII AR5 concludes that ‘…*the nature and extent of the impacts of implementing bioenergy depend on the specific system, the development context, and on the size of the intervention*’ (Smith *et al*., 2014).

Different case studies have documented that expanding production of the crops most commonly used to produce bioenergy can affect local incomes, food security, land tenure or health in positive and negative ways and that the outcomes of bioenergy production can be unequally distributed (Tilman *et al*., 2009; Persson, 2014). Model‐based assessments have tried to integrate sustainability considerations, pointing out likely interactions between bioenergy and food prices as well as biodiversity and water use(Popp *et al*., 2011; Lotze‐Campen *et al*., 2014; Scharlemann & Laurence, 2014). However, the effects of bioenergy on livelihoods and the role of governance agreements in promoting or mitigating specific types of impact have not yet been included in modelling exercises (Ackerman *et al*., 2009; Lubowski & Rose, 2013; Creutzig *et al*., 2014; Smith *et al*., 2014). Furthermore, previous studies have concluded that more clarity about the relationships between bioenergy production, livelihoods and equity is still needed (Creutzig *et al*., 2013; Hodbod & Tomei, 2013; Hunsberger *et al*., 2014).

In the light of the urgent need for action on climate change (IPCC, 2014), persistent economic and social inequalities, and intensifying competition for land (Lambin & Meyfroidt, 2011; Haberl, 2015), there is a need for science‐based policymaking with respect to the impacts of bioenergy on sustainable development. We have examined the scientific evidence base for such policymaking in a comprehensive systematic review using the scientific literature produced in the time period covered by the IPCC Fifth Assessment Report.

## Methodology for reviewing impacts of bioenergy production on sustainable development

The aim of this systematic review was to analyse the state of knowledge about how the production of bioenergy resources affects sustainable development. This is a key for understanding to what extent the existent knowledge can provide advice for policymakers. The systematic review focuses on the following impact categories: social, economic, institutional, environmental and technological (including food security and human health as social). The review is based on the assumption that if production of a bioenergy resource impacts any of the focus categories, it also impacts sustainable development. Thus, analysing the reported impacts on these focus categories will facilitate an overview of the state of knowledge regarding the impacts from bioenergy production on sustainable development.

We followed the steps included in the methodological guidance for systematic reviews by (Petticrew & Roberts, 2008; Bartolucci & Hillegass, 2010). The review protocol that served as methodological basis included five steps: (i) definition of scope and aims, (ii) research questions, (iii) search for and selection of evidence, (iv) quality appraisal and (v) data extraction and synthesis (see detailed protocol of the systematic review in the supplementary material).

We investigated to what extent the scientific community has answered the following questions which are of high interest in various contexts, including policy, in which decisions on future implementation of bioenergy are decided upon: Where do sustainable development impacts from bioenergy production take place? What is the evidence for the purported impacts? How are impacts attributed and measured? Are there certain context conditions that enable the observed impacts? Are the reported impacts specific to particular biomass resources? These questions were motivated by the discussions addressed in AR5, WGIII (Smith *et al*., 2014; annex on bioenergy). Although the AR5 considers impacts on sustainable development, it does not provide a geographically differentiated analysis or an understanding of the relation between context conditions and impacts. Several authors (Bustamante *et al*., 2014; Creutzig *et al*., 2014; Smith *et al*., 2014; von Stechow *et al*., 2015) explicitly highlight the need for improving the understanding of regional distribution of mitigation impacts on sustainable development, disaggregating by technologies and bioenergy inputs and under consideration of context conditions. The aim of this article was to make a first step in this direction through a stringent systematic review.

We used the same time frame for scientific publications as the Fifth IPCC Assessment report (AR5) (see supplementary information for the selection criteria and process) and went into a far more detailed analysis with regard to the questions reported above.

The AR5 defines bioenergy as ‘*energy derived from any form of biomass such as recently living organisms or their metabolic by‐products*’ (Allwood *et al*., 2014). We include nine biomass resources in the review: forest residues, unutilized forest growth, dedicated biomass forest plantations, combined forest sources, agriculture residues, dedicated biomass agricultural plantations, organic waste, combined agricultural resources and combined forest and agricultural resources (see protocol in the supplementary information for specific definition of each biomass resource). As the focus of the research was to understand the impacts from production and collection of these biomass resources on development, we did not distinguish the technologies used for producing bioenergy from biomass (i.e. first or second generation) but considered the demand that both technologies can create on biomass resources.

We acknowledge that there is no general agreement on how to measure impacts on sustainable development (Sneddon *et al*., 2006; Muys, 2013). Thus, we based the systematic review on the development impacts as outlined in the Agriculture, Forestry and Other Land Use (AFOLU) chapter of the IPCC WGIII AR5 (Smith *et al*., 2014). We considered a set of 33 potential impacts on sustainable development structured into five impact categories: institutional, social and health‐related, environmental, economic and technological (see Tables S3 and S4). We assumed that if production of a bioenergy resource affects any of these impact categories, it also affects sustainable development. Thus, analysing the reported impacts in a systematic manner provides an overview of the state of knowledge regarding how bioenergy production affects sustainable development as defined above.

### Selection of studies and data extraction

The selection process was carried out in three steps: definition of search criteria, a search in two scientific collections and a quality appraisal. For the search criteria, we included thirty inclusion criteria covering all five development categories and two further criteria on bioenergy forms for a set of sixty inclusion criteria combinations; and we included 12 exclusion criteria (see ‘article selection and data extraction’ in the protocol included in the supplementary information for further details). We further refined the selection using 31 categories of Web of Science, including 12 research areas. We limited the search to articles in English. The search was conducted in the Web of Science and in Science Direct including all their databases. This procedure yielded a wide and inclusive sample of 1175 articles covering all five development categories. For the quality appraisal, we randomly selected a subset of articles (*n* = 873 or 74.3% of the original sample), which makes the subsample representative. Only 541 of these passed the quality appraisal (criteria and procedure for the appraisal is clarified in the ‘quality appraisal’ section in the protocol included in the supplementary information). A total of 408 articles of the 541 (75.4%) were randomly included in the data extraction, and the research team carefully reviewed all articles. During the data extraction, we removed 92 articles because none of the 33 potential impacts included in our list were discussed, although they did discuss issues belonging to the five categories (that explains why these articles passed the quality appraisal). Thus, the results presented below are based on the analysis of the detailed data extracted from 316 original research articles that discuss at least one of the 33 impacts included.

### Data analysis

We analysed the data in three steps: (i) characterization of the study, (ii) consideration of the context conditions in the area of the study and (iii) reported impacts. Exploratory data analysis revealed a vast heterogeneity of how data were gathered, impacts attributed and results reported in the 316 analysed articles (see detailed counting of results in the supplementary information, file impacts trees). This heterogeneity combined with the number of variables mostly precluded the use of sophisticated statistical analysis methods, and our analysis is mainly based on descriptive tables and cross‐tabulations, combining data from all three steps. The statistical significance of potentially interesting relations between context conditions and impacts was analysed using Fisher tests (R Core Team, 2014).

## Results

Almost half of the articles in the systematic review analyse impacts from dedicated biomass plantations (agriculture and forestry), while few articles examine the sustainable development impacts from using agricultural or forestry residues (4 and 6%, respectively), or organic waste (2.5%) (see Table S10). Although several studies report that the use of organic waste as bioenergy feedstock can be associated with positive or low negative impacts, and hence considered an attractive bioenergy resource (Gregg & Smith, 2010; Haberl *et al*., 2011; Odlare *et al*., 2011), but the evidence in our review is insufficient to object or support this proposition as too few studies analyse this resource.

### Different places, different state of knowledge

Our results show an uneven geographical distribution of the studies, with most articles focusing on developed regions: 26.7% on Europe and 26.3% on North America; compared to only 13.1% on Asia, 8.2% on Africa, 7.8% on Latin America (Central and South America), 2.2% on Oceania; 15.7% of the studies conduct global analyses (Fig. 2, Table S11). This distribution contrasts with the share of annual plant biomass production (approximated through Net Primary Production or NPP) of these regions: 16% in Europe, 12% in North America, 19% in Asia, 20% in Africa, 26% in Latin America and 6% in Oceania (Krausmann *et al*., 2013). Although a multitude of socioeconomic and natural factors influences any region's technical or economic bioenergy potential, we consider NPP a useful proxy for its biophysical suitability for biomass production (Haberl *et al*., 2013). Modelling and empirical data suggest that current NPP levels may underestimate achievable productivities in human‐managed systems (DeLucia *et al*., 2014), but should be viewed in the perspective of scales of cultivation required for bioenergy to make an important contribution to the future energy supply and also possible ecological impacts of high‐input cultivation systems (Haberl, 2016).

Table 1 is divided into three categories of countries: (i) well‐studied key countries, (section A in Table 1); (ii) potentially relevant but understudied countries, that is, countries with high NPP but few, if any, studies (section B in Table 1); and (iii) relatively overstudied countries, that is, countries with low NPP and hence a relatively minor global contribution to the global bioenergy potential but nevertheless with many studies associated with them (section C in Table 1).

**Table 1:** Relation of studies and NPP values

Country	No. of studies	% of global NPP	Rank no. studies	Rank NPP
A. Countries with more than 1 study and more than 1% of global NPP				
United States	80	6.50	1	3
Brazil	25	12.10	2	1
China	13	5.60	4	5
India	13	2.30	5	10
Canada	9	6.00	10	4
Indonesia	9	3.20	12	8
United Republic of Tanzania	8	1.10	14	19
Australia	7	4.90	15	6
B. Countries with <5 studies and more than 1% of global NPP				
Russian Federation	3	11.30	27	2
Argentina	3	2.40	23	9
Dem. Rep. of the Congo	0	3.70	98	7
Colombia	0	1.90	89	11
Peru	1	1.60	51	12
Angola	0	1.50	65	13
Mexico	1	1.50	48	14
Venezuela	0	1.50	209	15
Bolivia	0	1.40	78	16
Sudan	0	1.30	192	17
Kazakhstan	0	1.20	131	18
C. Countries with 5 or more studies and <1% of global NPP				
Italy	14	0.24	3	63
Sweden	13	0.36	6	50
United Kingdom	12	0.23	7	65
Malaysia	10	0.56	8	32
South Africa	10	0.63	9	28
Germany	9	0.37	11	46
Thailand	9	0.51	13	35
Mozambique	6	0.91	16	22
Austria	5	0.08	17	97
Belgium	5	0.04	18	125
Spain	5	0.37	19	48
Denmark	4	0.05	20	119
France	4	0.58	21	31
the Netherlands	4	0.04	22	123

Net primary production (NPP) values calculated based on Haberl *et al*., (2011). For this table, we counted studies specialized in one country and studies looking at multiple countries, which are considered otherwise as global or regional studies. ‘Studies’ refers to the articles included in this systematic review.

The small share of studies considering impacts on sustainability in developing regions is surprising, as studies assessing global bioenergy potential commonly point to some of the countries in section B as possible large future suppliers of biomass and biofuels (Hoogwijk *et al*., 2009; Smeets & Faaij, 2010; Beringer *et al*., 2011; Haberl *et al*., 2011; Nijsen *et al*., 2012). For example, in Latin America, only Brazil (contributing 26 cases or 74% to the studies in countries of this region) emerges as a focal point of the scientific literature, while the number of country‐specific studies in other countries is small (three studies in Argentina and one study each in Costa Rica, Ecuador, Guatemala, Mexico and Peru). Hence, of the 20 countries in Latin America, only one country with a large NPP is well‐studied, whereas six countries are under‐studied despite their large potential. Extrapolations of impacts from the local/national to the regional level are thus not yet possible.

**Figure 1:**
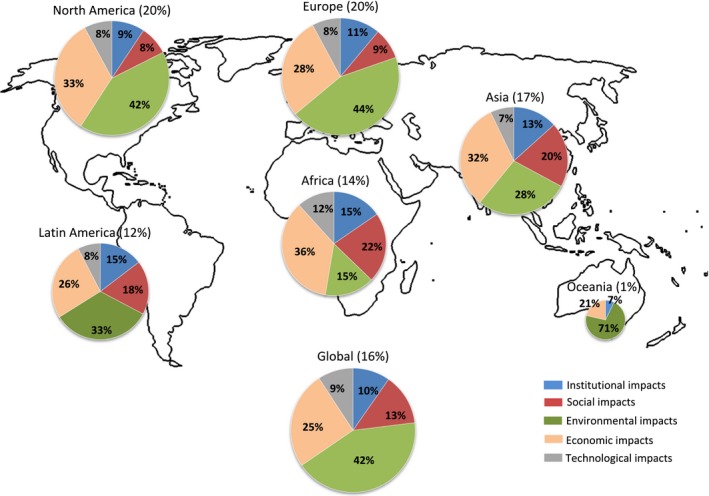
Regional distribution of the analysed impacts, reported as fraction of impacts within each category of all impacts analysed in each region. Percentage numbers after the region's name indicate the share of this region in the total of impacts considered and determine the size of the circle. Percentage numbers in the pies indicate the share of impacts each category contributes to the total number of impacts reported in the respective region. For all regions, the most reported social impact is food security; all other social impacts follow far behind. The outline map is from http://www.zonu.com/images/0X0/2009-11-05-10853/World-outline-map.png.

When looking at which impacts have been considered and where, our results show that most regions focus on the environmental and economic categories and barely consider social impacts with the exception of food security (see Fig. 1 and Table 2). Only studies focusing on Asia and Africa show a more balanced interest across categories.

**Table 2:**
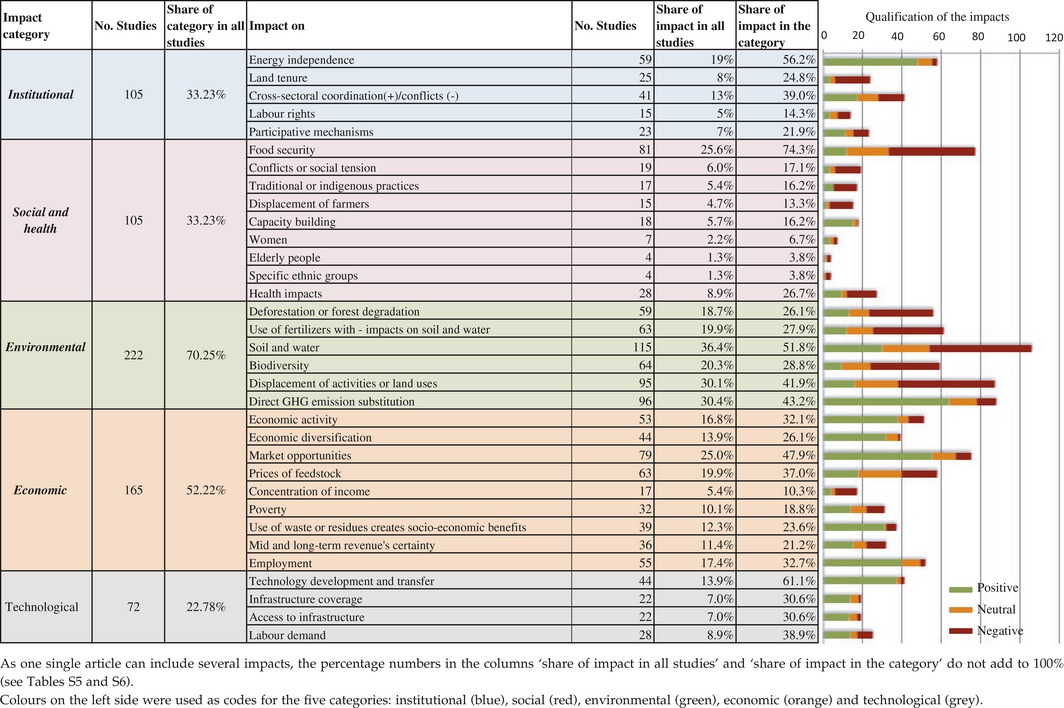
Distribution of analysed impacts per category

### Only a small number of impacts have been studied across regions

Beyond the impact categories, we further analysed which specific impacts were most frequently considered in each region (see Table 3). Studies at the global level focus on impacts on displacement of activities, on deforestation or forest degradation, on soil and water, on food security and on GHG emissions. To a lesser extent, but nevertheless important, global studies look at market opportunities, feedstock prices and technology development and transfer.

**Table 3:**
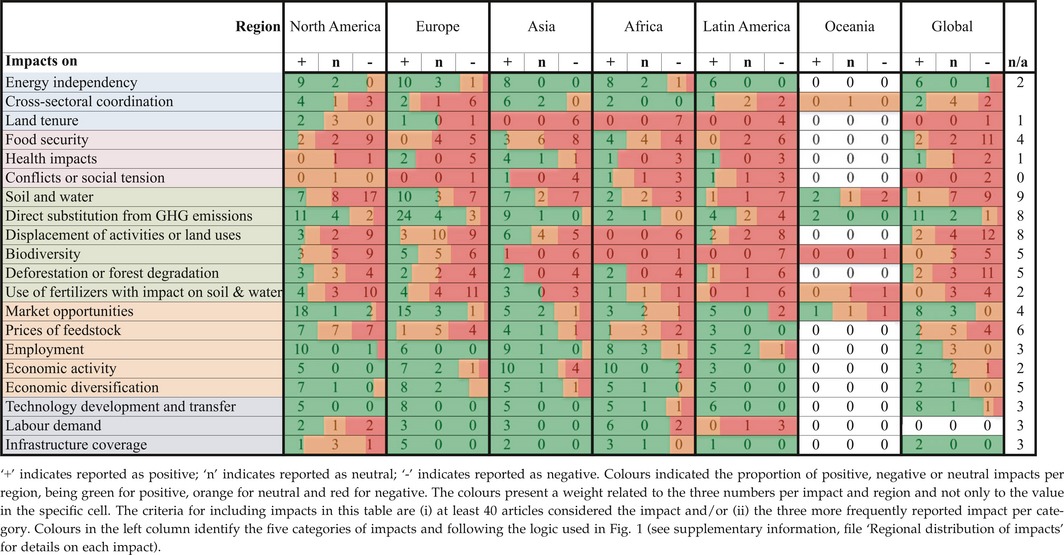
Positive and negative impacts per region

The regional distribution of the interest in specific impacts is uneven. In North America (mainly USA), impacts from the environmental category are included among the seven most frequent followed by impacts on prices of feedstock and on market opportunities from the economic category. The three most frequently analysed impacts in Europe and Latin America (mainly Brazil) are those on displacement of activities, on soil and water and on direct substitution of GHG emissions from fossil fuels. Studies from Oceania only consider six impacts: four of them in the environmental category with the most frequently analysed being impacts on soil and water.

The distribution of analysed impacts in Africa and Asia is more balanced. Most of the impacts have been considered in these two regions, suggesting a better engagement with the complexity of understanding sustainability impacts or an expectation that social impacts are relatively more important in these regions. The five impacts most often considered in Africa are impacts on food security, on energy independence, on economic activity, on employment and on poverty (in this order). In this region, impacts on *land tenure*, on *women* and on *capacity building,* are considered more often than in other regions. The five impacts most frequently considered in Asia are those on food security, on economic activity, on soil and water, on displacement of activities and on employment.

### Unbalanced understanding about impacts on sustainable development

The perspective of whether impacts are positive, negative or neutral is also uneven across regions. Our analysis of a selection of impacts shows that mostly negative impacts are reported in Latin America and at the global level, while the other regions show a more balanced picture (see Tables 3). The more detailed analysis presented below shows interesting differences in the importance given to each category and on where specific impacts were assessed as positive or negative.

Institutional impacts are included in over 30% of the articles (see Table 2). Within this impact category, *energy independence* is the most frequently studied impact across regions, especially in Europe and Africa, and biofuel deployment is reported mostly as having a positive impact on it. Other impacts in this category such as *cross‐sectorial coordination* show mixed results for all regions, while *land tenure* was reported as negatively impacted in Africa, Asia and Latin America.

**Figure 2:**
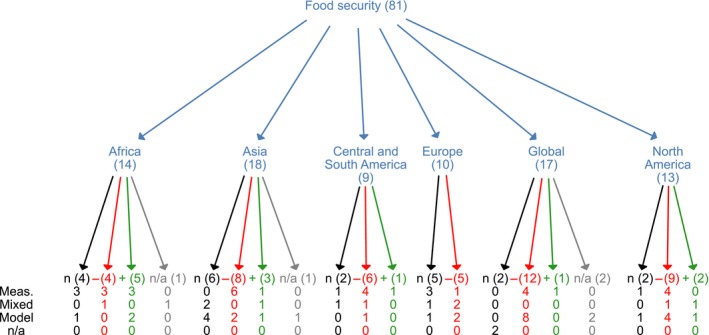
Impacts tree regarding food security. The blue arrows show the geographical distribution of the impacts on food security per regions as considered in the studies. In this case, there were no studies considering food security in Oceania. The first line indicates the number of positive (marked in green), negative (marked in red) or neutral impacts (marked in black). When the article did not specify the qualification of the impact, we considered it as nonavailable (n/a, marked in grey). From the second line downwards, we present how these impacts were identified, either using measurements, models or a combination (mixed). When the method was not clear in the article, we defined it as nonavailable (n/a). Impact trees for all other impacts considered in this systematic review are included in the supplementary information.

Social impacts are considered in over 30% of all studies, with *food security* being the most frequently addressed impact in this category (over 25% of the total studies and almost 75% of the articles considering social impacts). We undertook a detailed analysis of food security because it has been mentioned as one major concern for promoting deployment of bioenergy. Negative impacts on food security were reported twice as often as positive impacts. For all regions, impacts on food security are reported more often as negative than as positive, except in Africa where an equal number of studies report impacts as positive, negative or neutral (see Fig. 2 and Table 3).

In addition, we found that at the global level, the more often models are used for analysing impacts on food security, the higher the frequency of negative impacts (see Fig. 2). Although the small number of studies does not provide statistic robustness, this finding suggests a difference in the way impacts on food security are modelled or measured at the global level.

Other key social impacts – including gender and intragenerational impacts, social conflicts, displacement of farmers and impacts on traditional or indigenous practices – are insufficiently studied in all regions and practically not considered in global studies.

The environmental impact category is the most frequently considered category by the studies in the sample (over 70% of the total articles in the review, see Table 2), and each individual impact is addressed by at least a quarter of the studies. Across regions, all impacts in this category are reported as mostly negative or neutral, with the exception of *direct substitution of GHG emissions from fossil fuels,* which is considered positive or neutral in all geographical contexts. It is important to note, however, that over 65% of the studies used models for attributing direct substitution of GHG emissions from fossil fuels, and only 20% of these combined models with case study measurements. Thus, the qualification of this impact is highly dependent on the system boundaries and attribution criteria used. Negative impacts on the *displacement of activities or other land uses* are more frequently reported in Latin America, North America, Europe and at the global level (see Table 3). In Asia, slightly more positive impacts are reported compared to other regions.

Impacts on *biodiversity* are predominately reported as negative or neutral (see Table 3), except in a few studies from Europe and North America, whereas impacts on *deforestation or forest degradation* seem to be more negative for Latin America and at the global level. Further, impacts from the *use of fertilizers on soil and water* are reported as negative for Europe, North and Latin America, where these account for the majority of studies addressing this issue.

Economic impacts are considered in over half of all articles (see Table 2) and were predominantly positive for most impacts assessed in this category. Positive effects on *market opportunities* are noticeably reported in studies for North America and Europe (see Table 3), whereas positive effects on *economic activity* were more frequently reported in Africa and Asia. Impacts on *prices of feedstock* show mixed results for all regions. As for other impacts where modelling was used far more often than case study measurements, the positive or negative character of the economic impacts category needs more analysis considering the system boundaries and attribution criteria used.

Over 20% of all articles consider technological impacts (see Table 2). *Technology development and transfer* is the most frequently considered impact, followed distantly by *impacts on labour demand*,* infrastructure coverage and access to infrastructure*. Impacts on technology development and transfer are seen mostly as positive in all regions with only two studies reporting negative impacts: one from Africa and one at the global level (see Table 3).

### How context conditions influence development outcomes remains unclear

We analysed how impacts have been attributed by examining whether context conditions were explicitly reported. Context conditions describe the situation in the absence of additional biomass production and use for energy. Insight into these conditions is necessary for establishing a baseline or reference scenario and/or for attributing impacts on sustainable development from bioenergy production in a transparent manner. The systematic review includes 31 possible conditions that can describe the context in relation to the five impact categories (see supplementary information for a complete list of context conditions). We first analysed the extent to which impacts reported in the articles match to the corresponding context conditions at the level of category (i.e. whether context conditions were reported for those categories where impacts were identified).

**Figure 3:**
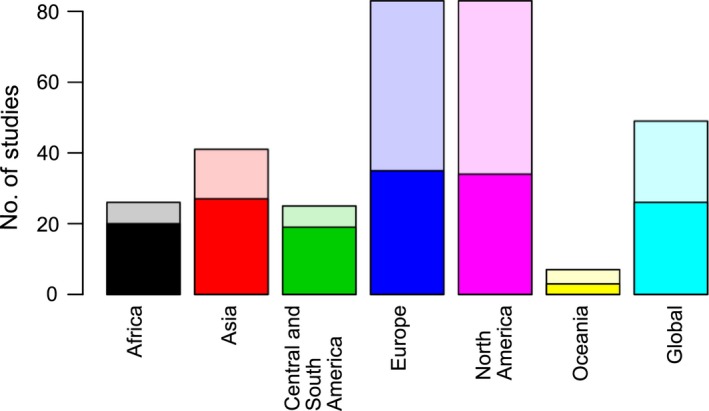
Geographical distribution of studies differentiating between studies considering or not considering context conditions. Solid colours indicate the number of studies with fully or partially matching context conditions. Transparent colours indicate the number of studies where context conditions were either not mentioned or do not correspond to the impact categories.

The analysis shows that only 13.6% of the articles comprehensively describe the context conditions against the category of the reported impacts, whereas 23% do not report context conditions at all. For the remainder, conditions were partially or fully mismatched (i.e. context conditions are described but not for the category of impacts reported). This lack of clarity of the context conditions applies to articles dealing with developed and developing countries, as well as global analyses. However, we found that studies analysing bioenergy production in developing countries report context conditions more often than studies on Europe, North America or those with a global scope (see Fig. 3). The lack of information applies across all reported impacts. For instance, from those articles quantifying impacts on food security, only 35% provide context conditions in the corresponding social category; concerning GHG emissions only 12% of articles provide corresponding baseline conditions. We recognize that for some standardized methodologies (e.g. LCA), and for most models, certain assumptions regarding context conditions are embedded in the procedures used. However, when they are not reported and/or validated, which is often the case, it remains unclear how impacts were attributed.

**Figure 4:**
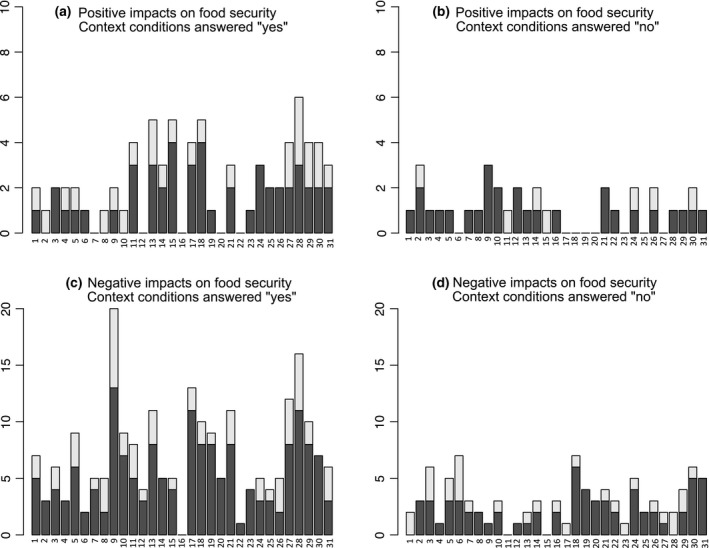
Impacts of bioenergy on food security related to the context conditions considered in this review. Y axis refers to number of articles, and X axis refers to context conditions following the numbering below. Dark grey shows the impacts attributed to dedicated agricultural crops, and hell grey indicates impacts attributed to any other biomass resource. Numbers in axis x numbering: (1) general conditions described. Institutional conditions: (2) the majority of households have access to energy; (3) land tenure clarified; (4) landscape management plan exists; (5) landscape policies exist and are enforced; (6) participation mechanisms are in place; (7) mechanisms for sectorial coordination are in place; (8) existing and enforced labour rights legislation; social conditions: (9) existing deficit in food access and/or supply; (10) existing social conflicts; (11) population growth is expected; (12) awareness about indigenous knowledge; (13) existing social networks/stakeholder organizations; (14) high average human capacity and skills; (15) low average human capacity skills; (16) equity mechanisms are in place; (17) social inequity reported as existing before bioenergy production; natural conditions: (18) land is available for people living in the area; (19) water for agriculture/forestry is available for people living in the area; (20) drinking water is available to people living in the area; (21) land (use) competition previous any intervention is reported in the article; (22) air quality is reported as good; (23) high biodiversity index. Economic conditions: (24) availability of capital; (25) existing crediting mechanisms; (26) sharing mechanisms of economic benefits in place; conditions related to technology and infrastructure: (27) traditional technologies; (28) modern (industrial) technologies; (29) combination of modern and industrial technologies; (30) technology is available to major local stakeholders; (31) mechanisms for technology development and/or transfer given.

We undertook a deeper analysis of the relationship between context conditions and several specific impacts. Initially, we conducted a descriptive analysis of impacts on food security, which is the most frequently reported social impact, to determine whether it is possible to establish the context conditions that trigger positive or negative impacts on food security. About 80% of the articles mentioning impacts on food security include some description of the context conditions. We found that in articles reporting impact on food security, most context conditions are considered at least once (see Fig. 4) and that no particular context condition clearly stands out in relation to either positive or negative impacts (e.g. conditions that are most frequent in the food security analysis, such as the use of modern technologies, show up both for negative and positive impacts).

The general lack of correlation between context conditions and impact sign is also reflected in the *P*‐values of Fisher tests, which we applied to all 1023 combinations of context conditions and impacts to check the influence of a particular context condition given or not given on the counts of impact signs. Table 4 displays that only 5 combinations have a *P*‐value below 5% and reports their corresponding numbers of condition–impact combinations.

**Table 4:** Combinations of conditions and impacts with *P*‐value below 5% in the Fisher test

Impact	Condition	*P*‐value (Fisher test)	Combination condition/impact					
			Yes/+	Yes/−	Yes/n	No/+	No/−	No/n
Food security or food production (negative if reduced or positive if improved)	Existing deficit in food access and/or supply	0.00154111	2	20	3	3	1	4
Conflicts or social tension	Existing deficit in food access and/or supply	0.02222222	7	1	2	0	0	1
Direct substitution of GHG emissions reductions from fossil fuels	Sharing mechanisms of economic benefits in place	0.03571429	0	2	0	6	0	0
Prices of feedstock	Modern (industrial) technologies	0.04449388	11	4	13	1	2	0
Employment (being employment creation (+) or employment reduction (−))	Mechanisms for sectorial coordination are in place	0.04545455	7	0	0	2	1	2

The Fisher test indicates whether the counts of impact signs in case of condition being ‘yes’ differ significantly from the counts of impact signs when the condition is ‘no’. Thus, a low *P*‐value does not represent strong evidence that the condition has an influence on the impact. This influence can only be postulated if the combination of conditions and impact also suggests its existence and direction. This is the case for only two combinations:

- Combination 1: context condition ‘existing deficits in food access and/or food security’ and impact on ‘food security’: when the context condition ‘existing deficits in food access and/or supply’ is given, then biomass production for bioenergy is almost exclusively reported to have a negative impact on food security. Studies reporting the absence of these deficits, on the other hand, report either a positive or a neutral impact on food security.
- Combination 2: context condition ‘benefit sharing mechanism for economic benefits are in place’ and impact on ‘direct substitution of GHG emissions from fossil fuels’: the impact on direct substitution of GHG emissions from fossil fuel is largely positive when no benefit‐sharing mechanism for economic benefits is in place, while the presence of such mechanisms exclusively leads to this impact being negative.

For the other three combinations in Table 4, the number of impacts is very small if the condition is answered with ‘no’ and the distribution of impacts (positive, negative or neutral) is ambiguous. Thus, even if the condition being ‘yes’ suggests a positive impact sign in two of these cases, it is not known whether these conditions really influence the corresponding impacts.

The regional analysis for the two combinations that in total suggest a correlation between condition and impact are displayed in Table 5. Fisher tests showed no significant difference between ‘yes’ and ‘no’ answers for any region.

**Table 5:** Regional distribution of relevant condition‐impact combinations

Region/Combination	‘Existing deficit in Food access’ and ‘Food security’							‘Sharing mechanisms in place’ and ‘Direct substitution of GHG emissions reductions’						
	Yes/+	Yes/−	Yes/n	No/+	No/−	No/n	Total	Yes/+	Yes/−	Yes/n	No/+	No/−	No/n	Total
Africa	1	2	2	1	0	0	6	0	0	0	0	0	0	0
Asia	1	6	1	0	0	2	10	0	0	0	2	0	0	2
Europe	0	2	0	0	0	1	3	0	0	0	1	0	0	1
North America	0	4	0	1	1	0	6	0	0	0	2	0	0	2
Oceania	0	0	0	0	0	0	0	0	0	0	0	0	0	0
Latin America	0	1	0	1	0	1	3	0	2	0	0	0	0	2
Global	0	5	0	0	0	0	5	0	0	0	1	0	0	1
Total	2	20	3	3	1	4	33	0	2	0	6	0	0	8

### Patterns in the distribution of positive and negative impacts

The results show some general patterns that are worth highlighting (see especially Figs 2, 3, 4 and Table 3). Impacts on some economic and technological categories are persistently positive across studies and regions. Within these categories impacts on energy independence, direct substitution of GHG emissions from fossil fuels, market opportunities, economic activity and diversification, employment as well as different technological categories is far most often reported as positive. In contrast, most impacts in the social and environmental categories are reported largely as having negative impacts, especially on land tenure, food security, displacement of other activities, biodiversity loss, and conflict and social tension. These patterns indicate an important trade‐off: that bioenergy projects may generate positive economic impacts but negative environmental and social impacts.

The incomplete information on context conditions (Fig. 3 and statistical analysis) makes it difficult to say anything conclusively across studies on what are the most relevant conditions triggering any specific impact. Yet, previous work has pointed to some reasons worth highlighting, notably that government institutions in countries targeted for bioenergy production often face severe constraints in implementing public policies and regulations intended to protect, for instance, land rights and food security (Ravnborg *et al*., 2013; Larsen *et al*., 2014). This is reinforced by our findings on context conditions related to food security and to some extent by the participation of governance‐related conditions highlighted through the Fisher Test. It is also worth noting that because climate change mitigation has been an important motivator for promoting bioenergy, it has been a higher research priority than other goals such as those related to biodiversity or land tenure. The latest IPCC Assessment Report made a great advance in including ethics and sustainable development in its considerations and paves the way for a more systemic research approach towards understanding development impacts from bioenergy production. More research is needed in the future to develop this approach, given the knowledge gaps identified in this review.

## Conclusions and outlook

Understanding the impacts of bioenergy production on sustainable development has been an important research topic in recent years, but its coverage is uneven, both in terms of geographical coverage, feedstocks considered, and in the categories of impacts considered. Furthermore, results are hardly comparable because context conditions and attribution criteria are not properly reported in the majority of the studies.

In the following, we present our conclusions about the research questions in this review.

### Where do sustainable development impacts from bioenergy production take place?

Geographically, we identified three distinct groups of countries, based on NPP as a proxy for biophysical biomass production potential, for considering bioenergy deployment in a given country. In the first group, we find countries with a high biophysical potential and a reasonable number of studies. These studies give good information about environmental and economic impacts, showing a tendency towards positive impacts from bioenergy production on direct substitution of GHG emissions from fossil fuels, market creation, technology development and transfer. However, social, institutional and technological impacts remain uncertain because they were far less often considered. The second group comprises countries with a high NPP but very few studies. Most of these are developing countries where there is a need for better understanding of possible sustainable development impacts of bioenergy implementation. For countries in this group, more research is needed to provide robust information for policymaking and governance agreements. The third group comprises countries with a relatively smaller NPP but many studies. This group consists mainly of developed countries and lessons on methodological issues from these studies can be used for future research in understudied countries.

### What is the evidence for the purported impacts and how are impacts attributed and measured?

There is a lack of systematic reporting on criteria for attributing impacts. Despite the existing discussion on attribution of specific methodologies (e.g. Finkbeiner, 2013; Muñoz *et al*., 2015 on attribution of indirect land use change in LCA), this omission in the studies makes it impossible to pursue a consistent comparison of results. We found that the environmental and economic impact categories were more thoroughly studied, whereas far less is known about how bioenergy production will affect the social and institutional categories of sustainable development. Institutional and social impact categories are better considered in country‐level studies than in global studies. Although there is an apparent indication of trade‐offs between positive impacts on the economic category and negative impacts on the environmental and social categories, more clarity about what triggers the trade‐offs could not be achieved due to the noncomparability of the results across the studies (lack of attribution criteria) and to the lack of information on context conditions in the majority of the studies.

### Are there certain context conditions that enable the observed impacts?

We found that there is a gap on reporting the specific context conditions prior to any intervention aimed at producing biomass for bioenergy, with less than 15% of the studies providing a comprehensive presentation of the context conditions in the category on which they attributed impacts. The lack of consistency in reporting context conditions and their relation to the reported impacts prevents clear and definitive conclusions on how the context affects the development outcome. Previous assessments have highlighted the need for ‘good governance’ as a condition required for promoting positive impacts of bioenergy production (Creutzig *et al*., 2014; Hunsberger *et al*., 2014; Smith *et al*., 2014). The reported negative impacts on land tenure, food security and food production, or other social and institutional aspects bear witness that bioenergy deployment can result in undesirable consequences and on the importance of understanding the context conditions, especially existing governance of natural resources.

### Are the reported impacts specific to particular biomass resources?

We found a concentration of studies dealing with dedicated biomass production, especially agricultural plantations. Other biomass resources have been less studied, and the use of waste as bioenergy feedstock has not received much systematic scrutiny. We conclude that analytical frameworks and methods that facilitate the analysis at a higher level of complexity, that is, including more categories or allowing aggregation from various studies, are still needed. Such frameworks need to ask for the inclusion and reporting of context conditions, explicitly and transparently, so that context‐dependent differences can be identified. Future empirical research, especially case studies, should aim to inform about the most effective governance arrangements – and identify situations where governance agreements have insufficient capacity to guarantee that bioenergy deployment consider international due diligence standards.

It is opportune to interpret our results in the context of the recent IPCC assessment of climate change. The IPCC author team concluded that:

One strand of literature highlights that bioenergy could contribute significantly to mitigating global GHG emissions via displacing fossil fuels, better management of natural resources, and possibly by deploying BECCS. Another strand of literature points to abundant risks in the large‐scale development of bioenergy mainly from dedicated energy crops and particularly in reducing the land carbon stock, potentially resulting in net increases in GHG emissions (Smith *et al*., 2014)

One interpretation of this divergence is that the first strand of literature emphasizes technological opportunities, such as yield increases, to reduce land use impact, and reap economic opportunities, while the other strand of literature investigates environmental dimensions under risk of being harmed (Creutzig, 2014). The growing literature exploring sustainable landscape management systems for the provision of biomass and other ecosystem services might gradually come to bridge the gap between these two strands of literature. Not the least, the integration of bioenergy systems into agriculture landscapes has been recognized as a promising option for addressing environmental impacts associated with current agriculture systems (Clarke *et al*., 2014; Edenhofer *et al*., 2014; Smith *et al*., 2014).

The IPCC report annex on bioenergy also points out that environmental, social and economic consequences of bioenergy deployment are site specific, but remains inconclusive on weighting the consequences across case studies. This review goes beyond the IPCC assessment in providing a comprehensive meta‐analysis, demonstrating that case studies evaluated so far tend to see increased economic and employment opportunities, GHG savings from fossil fuel displacement, and infrastructure development, but also risks related to land use change, in particular GHG emissions, food security, soil and water quality, biodiversity, and socially problematic outcomes.

Since the publication of the latest IPCC assessment report, further research on bioenergy has been published, which is in line with the main conclusions of our systematic review. The screening of this literature suggests that case studies mostly emphasize GHG emissions metrics and economic performance (e.g. (García *et al*., 2015; Mandaloufas *et al*., 2015)) and Dale *et al*. (2015) point out the importance of appropriate sustainability criteria and indicators. This observation suggests that the systematic bias observed in our survey of case studies can be interpreted as showing that social dimensions have been assigned a lower priority by scientists and policy processes than some environmental and economic dimensions.

There are limitations to the systematic review presented in this article. First, the complexity of the subject of analysis, such as the high number of potential interactions within the system boundaries and the lack of inclusion of criteria for analysing trans‐boundary impacts or trade‐offs between specific criteria and scale of the impacts, renders results of models and case studies partially inconclusive and subject to *a priori* values of investigators (Tribe *et al*., 1976). Second, most results in both cases depend on attributional accounting, which has been argued to be possibly misleading, while consequential accounting, being subject to higher uncertainties, might provide more policy‐relevant information. This is especially relevant for studies using LCA methods (Brandao *et al*., 2013; Hertwich, 2014; Plevin *et al*., 2014a,b). Third, we focused on studies published in English only. These limitations should be considered in future studies and analysed using complementary assessment methods.

Overall, we find that comparatively assessing the impacts of bioenergy production on sustainable development using the available scientific literature is a considerable challenge, but we are able to propose four recommendations for future research: (i) pursue a more stringent use of frameworks and methodologies that attribute impacts of bioenergy production on all development categories, (ii) report context conditions and criteria for attributing development impacts transparently, (iii) improve understanding of impacts of bioenergy production in developing countries with potentially favourable biophysical conditions for bioenergy and (iv) improve understanding of potential sustainable development impacts in different regions of using other bioenergy feedstock than biomass from dedicated plantations (e.g. organic waste and/or agricultural/forestry residues). Addressing these issues is essential for providing a more solid scientific basis for policymaking and governance agreements in the field of bioenergy and sustainable development.

## Supporting information


**Data S1.** Bioenergy production and sustainable development: limited science base for policy making‐Protocol.
**Table S1.** Characterization.
**Table S2.** Conditions.
**Table S3.** Potential impacts.
**Table S4.** Possible answers to potential impacts.

**Data S2.** Additional results.
**Table S5**. Number of studies per category and impact (*n* = 316).
**Table S6.** Positive, negative and neutral impacts.
**Table S7.** Methodological approaches used.
**Table S8.** Methodological approach per impact considered.
**Table S9.** Number of articles per method.
**Table S10.** Cross‐analysis potential vs. bioenergy resource.
**Table S11.** Aggregated regional distribution of impacts.
**Table S12.** Country distribution of the impacts considered.

**Data S3.** Article selection and data extraction.
**Table S13.** Basic set of articles.
**Table S14.** Appraisal results.
**Table S15.** Set of studies included in the data extraction.
**Table S16.** Data extraction – Characterization.
**Table S17.** Data extraction – Conditions.
**Table S18.** Data extraction – Potential impacts.

**Data S4.** Regional distribution of each impact considered in the systematic review.
**Figure S1.** Regional distribution of impacts on energy independence.
**Figure S2.** Regional distribution of impacts on land tenure.
**Figure S3.** Regional distribution of impacts on cross‐sectoral coordination.
**Figure S4.** Regional distribution of impacts on labour rights.
**Figure S5.** Regional distribution of impacts on participative mechanisms.
**Figure S6.** Regional distribution of impacts on food security.
**Figure S7.** Regional distribution of impacts on conflicts or social tension.
**Figure S8.** Regional distribution of impacts on traditional or indigenous practices.
**Figure S9.** Regional distribution of impacts on displacement of farmers.
**Figure S10.** Regional distribution of impacts on capacity building.
**Figure S11.** Regional distribution of impacts on women.
**Figure S12.** Regional distribution of impacts on elderly people.
**Figure S13.** Regional distribution of impacts on specific ethnic groups.
**Figure S14.** Regional distribution of impacts on health.
**Figure S15.** Regional distribution of impacts on deforestation or forest degradation.
**Figure S16.** Regional distribution of negative impacts of fertilizers on soil and water.
**Figure S17.** Regional distribution of impacts on soil and water.
**Figure S18.** Regional distribution of impacts on biodiversity.
**Figure S19.** Regional distribution of impacts on displacement of activities or land uses.
**Figure S20.** Regional distribution of impacts on direct GHG emission substitution.
**Figure S21.** Regional distribution of impacts on economic activity.
**Figure S22.** Regional distribution of impacts on economic diversification.
**Figure S23.** Regional distribution of impacts on market opportunities.
**Figure S24.** Regional distribution of impacts on feedstock prices.
**Figure S25.** Regional distribution of impacts on concentration of income.
**Figure S26.** Regional distribution of impacts on poverty.
**Figure S27.** Regional distribution of impacts on the use of waste or residues creating socio‐economic benefits.
**Figure S28.** Regional distribution of impacts on mid and long‐term revenue's certainty.
**Figure S29.** Regional distribution of impacts on employment.
**Figure S30.** Regional distribution of impacts on technology development and transfer.
**Figure S31.** Regional distribution of impacts on infrastructure coverage.
**Figure S32.** Regional distribution of impacts on access to infrastructure.
**Figure S33.** Regional distribution of impacts on labour demand.
