# Supplementary material for: Bioenergy production and sustainable development: science base for policymaking remains limited
Source: Glob Change Biol Bioenergy. 2016 Mar 23;9(3):541–56. doi: 10.1111/gcbb.12338 (PMC5340281; doi:10.1111/gcbb.12338)
Supplement: Supplementary file 1 — Data S1. Bioenergy production and sustainable development: limited science base for policy making‐Protocol. Table S1. Characterization. Table S2. Conditions. Table S3. Potential impacts. Table S4. Possible answers to potential impacts. [file GCBB-9-541-s001.pdf]

Bioenergy production and sustainable development: limited science basefor policy making

Protocol

Prepared by Carmenza Robledo Abad and Hans-Jörg Althaus;
Comments by Esteve Corbera, Carol Hunsberger, Richard Plevin, Jürgen Reinhard and Christian Lauk

Revised version 27.03.2013
Agreed version 8.04.2013

This protocol documents the steps as taken in this systematic review. It was prepared following the guidance and recommendations for systematic reviews as in the scientific community (*1*, *2*).

Definition of scope and aims

The systematic review is aimed at analyzing the state of knowledge concerning effects on sustainable development caused by the production and collection of bioenergy inputs (crops, materials) in specific regions. The systematic review focuses on the following impact categories: social, economic, institutional, environmental, and technological (including food security and human health as a social). The review is based on the assumption that if production of a bioenergy resource[1] impacts any of the focus categories it also impacts sustainable development. Thus analyzing the reported impacts on these focus categories will facilitate an overview of the state of knowledge regarding the impacts from bioenergy production on sustainable development.

The sample was determined by the following criteria:

Topic = (deforestation OR livelihood* OR "environmental impact*" OR "social impact*" OR "land tenure" OR "land grabbing" OR participation OR "labor rights" OR "labour rights" OR "food security" OR "food insecurity" OR conflict* OR "population growth" or "indigenous people" OR equity OR "water scarcity" OR "land competition" OR biodiversity OR income OR "economic" OR "traditional technology" OR "modern technology" OR "technology transfer" OR "sustainable development" OR sustainability) AND Topic=(bioenergy OR biofuel) NOT Topic=(meteorology OR gaseous OR "greenhouse gas*" OR hydrogenation OR "carbon dioxide" OR aerobic OR pyrolysis OR microscope OR thermodynamic OR thermochemical OR "Fischer-Tropsch")

Refined by: Web of Science Categories = (ENERGY FUELS OR ENVIRONMENTAL SCIENCES OR AGRICULTURAL ENGINEERING OR ENVIRONMENTAL STUDIES OR ENGINEERING ENVIRONMENTAL OR INTERNATIONAL RELATIONS OR AGRONOMY OR

---

[1] In this systematic review we distinguish the following biomass resources: forest residues, unutilized forest growth, agricultural residues, dedicated biomass forest or agricultural plantations and organic waste.

LAW OR ECOLOGY OR FORESTRY OR NUTRITION DIETETICS OR ECONOMICS OR ETHICS OR AGRICULTURE MULTIDISCIPLINARY OR PUBLIC ADMINISTRATION OR FOOD SCIENCE TECHNOLOGY OR MULTIDISCIPLINARY SCIENCES OR PLANNING DEVELOPMENT OR BIODIVERSITY CONSERVATION OR SOCIOLOGY OR AGRICULTURAL ECONOMICS POLICY OR AREA STUDIES OR WATER RESOURCES OR HISTORY PHILOSOPHY OF SCIENCE OR GEOGRAPHY OR ANTHROPOLOGY OR GEOGRAPHY PHYSICAL OR SOIL SCIENCE OR POLITICAL SCIENCE OR PUBLIC ENVIRONMENTAL OCCUPATIONAL HEALTH OR GEOSCIENCES MULTIDISCIPLINARY OR TRANSPORTATION) AND Document Types=(ARTICLE) AND Research Areas=(ENERGY FUELS OR ENVIRONMENTAL SCIENCES ECOLOGY OR AGRICULTURE OR ENGINEERING OR FORESTRY OR BUSINESS ECONOMICS OR FOOD SCIENCE TECHNOLOGY OR PUBLIC ADMINISTRATION OR BIODIVERSITY CONSERVATION OR WATER RESOURCES OR GOVERNMENT LAW) AND Languages=(ENGLISH)

Timespan=2008-01-01 - 2013-04-17. Databases=SCI-EXPANDED, SSCI, A&HCI.

This gives a basic set sample of 1175 articles. Then we selected a subset of the these articles randomly (n= 873 articles = 74.30%) (see excel file "Article selection and data extraction" included in this supplementary information). The sheet "basic set" includes all 1175 articles; the sheet "Appraisal results" includes all 873 articles included in the appraisal and the results, the first 541 are those articles that passed the appraisal.

Research questions
The review aims to respond to the following questions:

1. Where do the impacts from bioenergy resource production on sustainable development take place?
2. What is the evidence for the purported impacts?
3. How are impacts attributed and measured?
4. Are there certain context conditions that enable observed impacts?
5. Are reported impacts specific to particular biomass resources?

Search for and selection of evidence
A systematic review shall keep strength criteria for selecting the evidence. Making exceptions will create inconsistencies (*2*, *3*) The selection of publications was done as follows:

1. Search in two scientific collections: Web of Science and Science Direct (all databases).
2. Out of the 1175 articles 74.3% (n=873) were included in the appraisal.

Quality appraisal
The aim of this step is to validate that all articles selected should be included in the review (*2*). Thus the quality appraisal consists of a fast assessment of the paper in order to determine if an article should be excluded. This appraisal will be done using

an excel table, previously prepared. That increases consistency, reduces bias and facilitates the work of the reviewers.

Procedure:

Criteria for article exclusion	Yes	No
The article is NOT a meta-analysis or a review of other articles (if the article is a meta-analysis the answer is NO)		
The article discusses at least one of the focus impact categories (i.a. social, economic…)		
The article deals with at least one of the biomass resource categories included in the systematic review (cf. Tab. 1)		

If any of the previous questions is answered with “no”, the article will be excluded from the review. Meta-analysis and reviews shall be excluded in order to avoid “double accounting” of a given article.

Rules:

- Articles are distributed randomly among co-authors.
- If a co-author of the review has participated in the specific article (as co-author or reviewer), she/he shall give the specific article back and will receive a new one instead.

Results: after the appraisal 541 articles passed the appraisal criteria (n=46.06% of the full sample and 61.97% of the sub-sample included in the appraisal). These 541 articles are the sub-set 2 used as basis for the data extraction, for which the data extraction was conducted (see excel file “Article selection and data extraction”, sheet Appraisal results.

Data extraction and synthesis
The data extraction was done on over 75.4% of articles out of the sub-sample and selected randomly (n=408 of the from the remaining article after the appraisal – sub-set 2) We used an Excel format previously prepared, where all criteria for the data extraction and the given options for answers are included (see in this supplementary material excel file “Article selection and data extraction” sheet Data extraction-set)

The time used for the data extraction was between October 28th, 2013 and July 15th, 2014. Each reviewer extracted the specific data per article according to the tables 1-4. These tables were included in an Excel format for facilitating the whole review.

Table SI1 presents the criteria and assessment values regarding characterization of the publication (see explanations below the table, see excel file “article selection and data extraction”, sheet data extraction and characterization for the results, and excel file included in this supplementary material).

Table SI1: Characterization

**Category**	**Aspect**	**Answer**
	Publication	Complete reference (given)
Reviewers	Reviewer's name	
Characterizatio n	Type	Options given (single answer):<br>- Method or framework description<br>- Case study (one or multiple)<br>- Combination of method/framework description and case studies<br>Additional space for comments provided (optional)
	Biomass resource category[2]	Options given (*4*, *5*)(multiple answers):<br>- Forest residues<br>- Unutilized forest growth<br>- Dedicated biomass forest plantations<br>- Combined forest sources<br>- Agriculture residues<br>- Dedicated biomass agricultural plantations<br>- Organic waste<br>- Combined agricultural resources<br>- Combined forest and agricultural resources<br>Additional space for comments provided (optional)
	Region	Options given (single answer):<br>- Global<br>- Africa<br>- Asia<br>- Europa<br>- North America<br>- Central and South America<br>- Oceania<br>Additional space for comments provided (optional)
	Country	A list of all UN countries will be given per region (single answer). An option “various countries” will be given for those cases when an article presents results from different countries. This option can be combined either with a region (when all countries are in the same region) or global (when the countries are in different continents)<br>Additional space for specific list of countries (optional)
	Measurement or model	Options given (single answer):<br>- Measurement (including current and/or past measurements)

[2] See section 10 for definitions of each category.

Category	Aspect	Answer
		- Model<br>- Mixed
	Assessment method	Options given (single answer):<br>- Qualitative<br>- Qualitative and explicit<br>- Explicit quantification<br>- Implicit quantification<br>Additional space for comments provided (optional)

Explanations:

Measurement or model?

Here we distinguish between three possible types of papers:

- Measurement/observation, i.e. existing cases are assessed based on qualitative or quantitative data and observations direct from the field (e.g. XX ha of native forest were cleaned for planting palm oil or local population reports abuse regarding land use).
- Model, i.e. impacts are calculated using models, based on assumptions or extrapolations in the model (e.g., scenario development, integrated assessment modeling, LCA)
- Mixed: impacts are calculated based on specific (for the study reported in the article) measurements or estimations, including historical data.

Assessment method:

The types of methods considered are:

1. Implicit qualitative: either descriptive or argumentative. These articles present qualitative results of impacts on sustainable development (potential or experienced) Assumptions or rules for attribution are not explicit

2. Qualitative and explicit: either descriptive or argumentative. In these articles impacts on sustainable development (potential or experienced) are presented/reported AND assumptions or rules for attribution are explained

3. Explicit quantification: when the paper:
    a. Presents a quantification of impacts on sustainable development, AND
    b. rules and assumptions for attributing impacts on sustainable development are explicitly clarified or documented in the given article (rules can be given as per norms, e.g. if an article says that the LCA was done following the ISO norm)

4. Implicit quantification: when the paper:
    a. Presents quantitative results of impacts on sustainable development, BUT
    b. Rules and assumptions for attributing impacts on sustainable development are NOT explicitly clarified or documented in the given article

Table SI2 below presents how context conditions were included in the review. Context conditions are conditions existing prior to an intervention.

Explanations:
There are three possible answers, yes, no or n/a (not -available). Answers (yes, no or not -available) are given according to what is explicitly reported in the article. That means:

"Yes" is used when the specific condition is reported as fulfilled.
"No" is used when the specific condition is reported as unfulfilled.
"not-available" is used when the article doesn't consider the specific condition.

Results from the context conditions are included in the excel file "Article selection and data extraction" sheet Data extraction-Conditions which is included in this supplementary material.

Table SI2: Conditions

	Context conditions	Yes	No	n/a
General conditions are mentioned (e.g. description of the area, some description of social group involved and their living conditions)				
Institutional	The majority of households have access to energy			
	Land tenure clarified			
	Landscape management plans exist			
	Landscape policies exist and are enforced			
	Participation mechanisms are in place			
	Mechanisms for sectorial coordination are in place			
	Existing and enforced labor rights legislation<br>Additional space for comments provided (optional)			
Social	Existing deficit in food access and/or supply			
	Existing social conflicts			
	Population growth is expected			
	Awareness about indigenous knowledge			
	Existing social networks/ stakeholder organizations			
	High average human capacity and skills			
	Low average human capacity and skills			
	Equity mechanisms are in place			
	Social inequity (any type) reported as existing previous any bioenergy production			
	Additional space for comments provided (optional)			
Natural	Land is available for people living in the area			
	Water for agriculture/forestry is available for people living in the area			
	Drinking water is available to people living in the area			
	Land (use) competition previous any intervention is reported in the article			
	Air quality is reported as good			
	High biodiversity index			
	Additional space for comments provided (optional)			
Economic	Availability of capital			
	Existing crediting mechanisms			
	Sharing mechanisms of economic benefits in place<br>Additional space for comments provided (optional)			
Technology and infrastructure	Traditional technologies			
	Modern (industrial) technologies			
	Combination of modern and industrial technologies			
	Technology is available to major local stakeholders			

	Mechanisms for technology development and/or transfer given			
	Additional space for comments provided (optional)			

Table SI3 presents the potential impacts on sustainable development per focus category while table 4 presents the corresponding data and values to be assessed per each potential impact. The co-authors were requested not to make a judgment, but to extract what and how is reported in the article.

Results regarding potential impacts are included in the excel file "Article selection and data extraction" sheet Data extr –Potential impacts, which is included in this supplementary material.

Table SI3: Potential impacts

**Category**	**Potential impacts on**
Institutional Issues	Energy independence
	Impacts on and tenure for local stakeholders
	Cross sectorial coordination (+) or cross-sectorial conflicts (-)
	Impacts on labor rights (recognition and/or empowerment) and conditions along the value chain
	Participative mechanisms for small-scale producers
	Additional space for comments provided (optional)
Social and health	Food security or food production (negative if reduced or positive if improved)
	Conflicts or social tension
	Impacts on Traditional or indigenous management practices
	Displacement of farmers
	Capacity building and new skills
	Women
	Elderly people
	Specific ethnic groups
	Health impacts
	Additional space for comments provided (optional)
Environmental	Deforestation or forest degradation
	Use of fertilizers with negative impacts on soil and water
	Soil and water
	Biodiversity
	Displacement of activities or other land uses
	Direct substitution of GHG emissions reductions from fossil fuels
	Additional space for comments provided (optional)
Economic	Economic activity and income per family/per household
	Economic diversification
	Market opportunities (+ if it increases opportunities and – if it

	decreases opportunities)
	Prices of feedstock
	Concentration of income (- if it promotes concentration and + if it promotes deconcentration of income)
	Poverty (being (+) if it contributes to reduces poverty or (-) if it increases poverty (
	Using waste and residues creates socio-economic benefits with little/no environmental risk
	Certainty about mid and long-term revenue
	Employment (being employment creation (+) or employment reduction (-))
	Additional space for comments provided (optional)
Technological	Technology development and transfer (being (+) when promoting it or (-) when disincentivating it
	Infrastructure coverage (
	Access to infrastructure
	Labor demand (New technology increase labor demand (+) or reduce labor demand (-))
	Additional space for comments provided (optional)

Table SI4: Possible answers to potential impacts

Is the potential impact considered in the article?	yes	no	
Is the impact considered positive, negative or neutral?	+	-	n
Do the authors use quantitative indicators?	yes	no	
Which indicators?			
Range of results?			

+ = Positive, - = Negative, n = Neutral

Methods for addressing the research questions

The research questions will be addressed using descriptive statistics. If possible (i.e. if the data basis is not too heterogeneous) t-tests (or U-tests, depending on the distribution in the sample) and / or cluster analysis. Descriptive statistics and cluster analyses will be used to enunciate hypotheses about correlations and differences between classes in the sample, which will be tested by t-tests (or U-tests). The combination of these methods has a further advantage for this systematic review as it allows an iterative process of knowledge discovery and a multi-objective optimization as a result of many rounds of trail and failure.[3]

Depending on the comparability of the data among all articles, the research questions will be broken down into more specific questions that allow identifying clusters of answers.

[3] Descriptive statistics will suggest correlations which can then be tested. It is not sensible to a priori hypothesize correlations before scanning data

Depending on the quality and comparability of the data basis one can expand the set of specific questions according to the evolution of the analysis. We will analyse the possibility to get different types of clustering (from hierarchical to density-based) that can be used according to the data extracted and to the specific questions. The detailed assessment steps will be documented.

Guidance for co-authors

All authors accept the following rules for collaboration:

- No one can review an article in which she/he has been included either as a co-author or a reviewer
- No one will promote own results or findings. This is a source of bias that shall be avoided in a systematic review.
- We commit with a schedule (see section 8). Some delays are understandable, but if a co-author doesn't provide feedback within a reasonable period the research can not continue. If there is a serious delay, without any communication to the lead author in sending the appraisals or the articles data the lead author can delete the name from the list of co-authors
- In principle we will try to achieve consensus. If disagreement remains, the lead author will prepare a proposal for a reasonable compromise. If no agreement can be achieved the authors will vote. Simple majority decides (n=50%+1). In the case of a standoff, the final ballot is with the lead author. If an author still disagrees, she/he can leave the publication. His/her name will then be included in the acknowledgments.

Access to data and assessment proceedings

The following material will be prepared:

1. Protocol
2. Excel files (2); one for the appraisal and one for the data extraction
3. Excel with data compilation (managed by the lead author)
4. Tables with the statistic assessment (managed by the lead author)
5. Drafts and final manuscripts.

All co-authors will receive/have access to all material.

List of co-authors

Robledo-Abad[1,2*], C., H.J. Althaus[3,4], G. Berndes[5], S. Bolwig[6], E. Corbera[7], F. Creutzig[8], J. Garcia-Ulloa[9], A. Geddes[10], J. S. Gregg[6], H. Haberl[11], S. Hanger[10, 22], R.J. Harper[12], C. Hunsberger[13], R. K. Larsen[14], Ch. Lauk[11], S. Leitner[11], J. Lilliestam[10], H. Lotze-Campen[15, 23], B. Muys[16], M. Nordborg[5], M. Ölund[21], B. Orlowsky[17], A. Popp[15], J. Portugal-Pereira[18], J. Reinhard[19], L. Scheiffle[15], P. Smith[20],

Affiliations

[1] Department of Environmental Systems Science, USYS TdLab, ETH Zürich, Universitaetstrasse 22, 8092 Zurich, Switzerland.
[2] Helvetas Swiss Intercooperation, Maulbeerstr. 10, CH-3001, Bern, Switzerland.
[3] Foundation for Global Sustainability (ffgs), Reitergasse 11, 8004 Zürich, Switzerland.
[4] Lifecycle Consulting Althaus, Bruechstr. 132, 8706 Meilen, Switzerland

[5] Department of Energy and Environment, Chlamers University of Technology, Gothenburg, Sweden
[6] DTU Management Engineering, Technical University of Denmark, Roskilde, Denmark
[7] Institute of Environmental Science and Technology, and Department of Economics & Economic History, Universitat Autònoma de Barcelona, Spain.
[8] Mercator Research Institute on Global Commons and Climate Change & Technical University Berlin, Germany.
[9] Institute of Terrestrial Ecosystems, ETH Zürich, Universitaetstrasse 22 8092 Zurich, Switzerland.
[10] Institute for Environmental Decisions (IED), Climate Policy Group, Universitätstrasse 22, 8092 Zurich, Switzerland.
[11] Institute of Social Ecology Vienna (SEC), Alpen-Adria Universitaet (AAU), Schottenfeldgasse 29, 1070 Vienna, Austria.
[12] School of Veterinary and Life Sciences, Murdoch University, South Street, Murdoch, Western Australia 6150, Australia
[13] Department of Geography, University of Western Ontario. London, Ontario N6A 5C2, Canada.
[14] Stockholm Environment Institute (SEI), Linnégatan 87D, 115 23 Stockholm, Postbox 24218, 104 51 Stockholm Sweden.
[15] Potsdam Institute for Climate Impact Research (PIK), PO Box 601203, 14412 Potsdam, Germany.
[16] Division of Forest, Nature and Landscape, University of Leuven (KU Leuven), Celestijnenlaan 200E box 2411, BE- 3001 Leuven, Belgium.
[17] climate-babel.org, Aarau, Switzerland
[18] Energy Planning Program, COPPE, Federal University of Rio de Janeiro, Centro de Tecnologia, Sala C-211, C.P. 68565, Cidade Universitária, Ilha do Fundão 21941-972 Rio de Janeiro, RJ, Brazil.
[19] Informatics and Sustainability Research Group, Swiss Federal Institute for Material Testing and Research, Empa, Ueberlandstrasse 129, 8600 Duebendorf, Switzerland
[20] Institute of Biological & Environmental Sciences, ClimateXChange and Scottish Food Security Alliance-Crops, University of Aberdeen, 23 St Machar Drive, Aberdeen AB24 3UU, Scotland, UK
[21] Centre for Environment and Sustainability – GMV, University of Gothenburg, Aschebergsgatan 44 Göteborg, Sweden.
[22] International Institute for Applied Systems Analysis, Schlossplatz 1, Laxenburg, Austria
[23] Humboldt-University zu Berlin, Unter den Linden 6, 10099 Berlin, Germany

*Correspondence to: Carmenza Robledo-Abad at carmenza.robledo@usys.ethz.ch

Other collaborators

During the two years that we needed for finalizing the systematic review, some collaborators withdrew. However we, the co-authors of the final article, acknowledge their participation and their inputs during the discussions on the protocol and the appraisal. They are:

- Omar Masera, Center for Ecosystems Research, National Autonomous University of Mexico (CIECO, UNAM)
- Yacob Mulugetta; Centre for Environmental Strategy, University of Surrey, Guildford GU2 7XH
- Roberto Schaeffner, Energy Planning Program, COPPE, Federal University of Rio de Janeiro, Centro de Tecnologia, Sala C-211, C.P. 68565, Cidade Universitária, lha do Fundão 1941-972 Rio de Janeiro, RJ, Brazil
- Richard Plevin, Institute of Transportation Studies, UC Davis 1605 Tilia St. (West Village), Rm 100, University of California, Davis, USA
- Rainer Zah, Quantis International, Switzerland

1 Schedule

Step	What	Who	When (deadline)
1. Preparation of a protocol			
	Preparation of the protocol	Lead author	13-Mar 2013
	Comments to the protocol	Co-author	18-Mar 2013
	Protocol agreed	Lead author	08-April 2013
2. Selection of articles			
	Search in 2 data banks	Lead author	28-Mar 2013
	Completeness check	all	18-Apr 2013
3. Quality appraisal			
	Distribution of appraisal format	Lead author	30-Apr 2013
	Distribution of articles among co-authors	Lead author	10-Apr 2013
	Appraisal done	all	10-Oct 2013
4. Data extraction			
	Distribution of the data extraction format	Lead author	28-Oct 2013
	Redistribution of articles (according to the result of the appraisal)	Lead author	28-Oct 2013
	Data extraction	all	July 18, 2014
5. Data analysis and synthesis			
	Data analysis	Carmenza Robledo & Boris Orlowski with support from Hans-Jörg Althaus, Anna Geddes and Johan Lilliestam	August 20, 2014
	Workshop	Carmenza Robledo, Hans-Jörg Althaus, Jürgen Reinhard, John Garcia, Jay Sterling Gregg, Simon Bolwig, Esteve Corbera, Sue Hanger, Johan Lilliestam, Carol Hunseberg Rasmus Kloeker Larsen, Joana Portugal	October 2^{nd,} 2014

	Preparation of the first draft	Lead author/comments from all	October 22, 2014
6. Reporting			
	Invitation by GCBB	Lead author	August 25.2015
	Paper submission	Lead author	August 26, 2015

Authorship list according to contribution
Corresponding author first then all co-authors in alphabetic order

Working definitions

Biomass resource category
1. Forest residues: Residues from silvicultural thinning and logging; wood processing residues such as sawdust, bark and black liquor; dead wood from natural disturbances, such as storms and insect outbreaks (irregular source). Residue removal rates need to be controlled considering local ecosystem including biodiversity, climate, topography, and soil factors. ILUC affects are mostly negligible but may arise if earlier uses are displaced or if soil productivity losses require compensating production. There is a near term trade-off in that organic matter retains organic C for longer if they are left on the ground instead of being used for energy.
2. Unutilized forest growth: The part of sustainable harvest levels (often set equal to net annual increment) in forests judged as being available for wood extraction, which is above the projected biomass demand for producing other forest products. Includes both biomass suitable for, e.g., pulp and paper production and biomass that is not traditionally used. The resource potential and mitigation benefit depend (besides fossil C displacement efficiency) on both environmental and socio-economic factors: the change in forest management and harvesting regimes due to bioenergy demand depends on forest ownership and the structure of the associated forest industry; and the forest productivity and C stock response to changes in forest management and harvesting depend on the character of the forest ecosystem, as shaped by historic forest management and events such as fires, storms and insect outbreaks.
3. Agriculture residues: Manure (given separately in parenthesis and not included in the agriculture residue potential); harvest residues (e.g., straw); processing residues (e.g., rice husks from rice milling). Similar environmental restrictions on harvest residue removal as for forests. ILUC effects and timing of C flows also similar, although the longer term soil C trade-off may be less than previously believed. Residues have varying collection and processing costs (in both agriculture and forestry) depending on quality and how dispersed they are, with secondary residues often having the benefits of not being dispersed and having relatively constant quality. Densification and storage technologies would enable cost effective collections over larger areas.
4. Dedicated biomass plantations: including annual (cereals, oil- and sugar crops) and perennial plants (e.g., switchgrass, Miscanthus) and tree plantations (both coppice and single-stem plantations, e.g., willow, poplar, eucalyptus, pine). Higher end estimates presume favourable agriculture development concerning

land use efficiency - especially for livestock production - releasing agriculture lands for bioenergy. Diets are a critical determinant, given the large land requirements to support livestock production. Large areas presently under forests are biophysically suitable for bioenergy plantations but such lands are commonly not considered available due to GHG, biodiversity and other impacts. Grasslands and marginal/degraded lands (uncertain extent and suitability) are commonly considered as available for bioenergy, but their use require careful planning and crop selection to avoid negative impacts concerning GHG balances, water availability, biodiversity, and subsistence farming and equity.
5. Organic wastes: Waste from households and restaurants, discarded wood products such as paper and demolition wood, and wastewaters suitable for anaerobic biogas production. Organic waste may be dispersed and also heterogeneous in quality but the health and environmental gains from collection and proper management through combustion or anaerobic digestion can be significant. Also must consider whether the waste had an alternative use that will need to be met from some other source.

**Dimensions**	**Issues included (non-conclusive list)**
Social and human assets	Population growth and migration, level of education, human capacity, existence and forms of social organization, individual skills, indigenous knowledge, cultural values, equity and health
Natural assets	Availability of natural resources (land, forest, water, agricultural land, minerals, fauna), GHG balance, ecosystem integrity, biodiversity conservation, ecosystem services, ecosystem productive capacity, climate change resilience and vulnerability
State of infrastructure and technology	Availability of infrastructure and technology, technology development, appropriateness, acceptance
Economic factors/assets	Credit capacity, employment creation, income, wealth distribution/distribution mechanisms, carbon finance, available capital/investments
Institutional arrangements	Land tenure and land use rights, participation and decision making mechanisms (e.g. through FPIC), sectoral and cross-sectoral policies, investment in research

Based on (*6*–*10*)

References

1. A. A. Bartolucci, W. B. Hillegass, in *Evidence-Based Practice: Toward Optimizing Clinical Outcomes*, F. Chiappelli, Ed. (Springer Berlin Heidelberg, 2010; http://link.springer.com/chapter/10.1007/978-3-642-05025-1_2), pp. 17–33.

2. M. Petticrew, H. Roberts, *Systematic Reviews in the Social Sciences: A Practical Guide* (2008; http://onlinelibrary.wiley.com/book/10.1002/9780470754887).

3. Liz Victor, Systemic Reviewing in the Social Science: Outcomes and Explanation. *ENQUIRE*. **1**, 12 (2008).

4. H. Chum *et al.*, in *IPCC Special Report on Renewable Energy Sources and Climate Change Mitigation*, O. Edenhofer et al., Eds. (Cambridge University Press, Cambridge, United Kingdom and New York, NY, USA, 2011).

5. F. Creutzig *et al.*, *GCB Bioenergy*, in press, doi:10.1111/gcbb.12205.

6. M. K. Macauley, R. A. Sedjo, Forests in climate policy: technical, institutional and economic issues in measurement and monitoring. *Mitig. Adapt. Strateg. Glob. Change*. **16**, 499–513 (2011).

7. R. Madlener, C. Robledo, B. Muys, J. T. B. Freja, A Sustainability Framework for Enhancing the Long-Term Success of Lulucf Projects. *Clim. Change*. **75**, 241–271 (2006).

8. J. Pretty, Agricultural sustainability: concepts, principles and evidence. *Philos. Trans. R. Soc. B Biol. Sci.* **363**, 447–465 (2008).

9. P. Smith *et al.*, in *In: Climate Change 2014: Mitigation of Climate Change. Contribution of Working Group III to the Fifth Assessment Report of the Intergovernmental Panel on Climate Change [Edenhofer, O., R. Pichs-Madruga, Y. Sokona, E. Farahani, S. Kadner, K. Seyboth, A. Adler, I. Baum, S. Brunner, P. Eickemeier, B. Kriemann, J. Savolainen, S. Schlömer, C. von Stechow, T. Zwickel and J.C. Minx (eds). Chapter 11* (Cambridge University Press, Cambridge, UK and New York, NY, USA, 2014), vol. IPCC Fifth Assessment Report, WG III.

10. C. Sneddon, R. B. Howarth, R. B. Norgaard, Sustainable development in a post-Brundtland world. *Ecol. Econ.* **57**, 253–268 (2006).
