# Supplementary material for: Bioenergy production and sustainable development: science base for policymaking remains limited
Source: Glob Change Biol Bioenergy. 2016 Mar 23;9(3):541–56. doi: 10.1111/gcbb.12338 (PMC5340281; doi:10.1111/gcbb.12338)
Supplement: Supplementary file 4 — Data S4. Regional distribution of each impact considered in the systematic review. Figure S1. Regional distribution of impacts on energy independence. Figure S2. Regional distribution of impacts on land tenure. Figure S3. Regional distribution of impacts on cross‐sectoral coordination. Figure S4. Regional distribution of impacts on labour rights. Figure S5. Regional distribution of impacts on participative mechanisms. Figure S6. Regional distribution of impacts on food security. Figure S7. Regional distribution of impacts on conflicts or social tension. Figure S8. Regional distribution of impacts on traditional or indigenous practices. Figure S9. Regional distribution of impacts on displacement of farmers. Figure S10. Regional distribution of impacts on capacity building. Figure S11. Regional distribution of impacts on women. Figure S12. Regional distribution of impacts on elderly people. Figure S13. Regional distribution of impacts on specific ethnic groups. Figure S14. Regional distribution of impacts on health. Figure S15. Regional distribution of impacts on deforestation or forest degradation. Figure S16. Regional distribution of negative impacts of fertilizers on soil and water. Figure S17. Regional distribution of impacts on soil and water. Figure S18. Regional distribution of impacts on biodiversity. Figure S19. Regional distribution of impacts on displacement of activities or land uses. Figure S20. Regional distribution of impacts on direct GHG emission substitution. Figure S21. Regional distribution of impacts on economic activity. Figure S22. Regional distribution of impacts on economic diversification. Figure S23. Regional distribution of impacts on market opportunities. Figure S24. Regional distribution of impacts on feedstock prices. Figure S25. Regional distribution of impacts on concentration of income. Figure S26. Regional distribution of impacts on poverty. Figure S27. Regional distribution of impacts on the use of waste or residues creating socio‐e [file GCBB-9-541-s004.pdf]

Regional distribution of impacts from “Bioenergy production and sustainable development: limited science base for policy making” by Robledo-Abad et al.

The following figures show the regional distribution of each impact considered in the systematic review. The first line indicates the number of positive (marked in green), negative (marked in red) or neutral impacts (marked in black). When the article didn’t specify the qualification of the impact we considered it as non-available (n/a, marked in grey). From the second line downwards we present how these impacts were identified, either using measurements, models or a combination (mixed). When the method was not clear in the article we defined it as non-available (n/a).

1 Institutional impacts

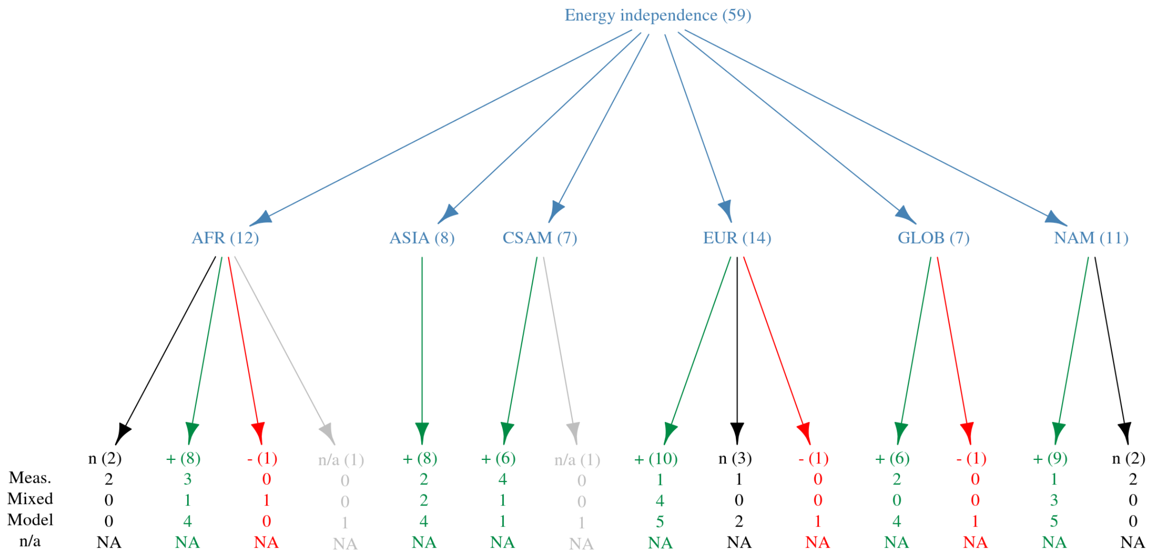

Figure S1: Regional distribution of impacts on energy independence.

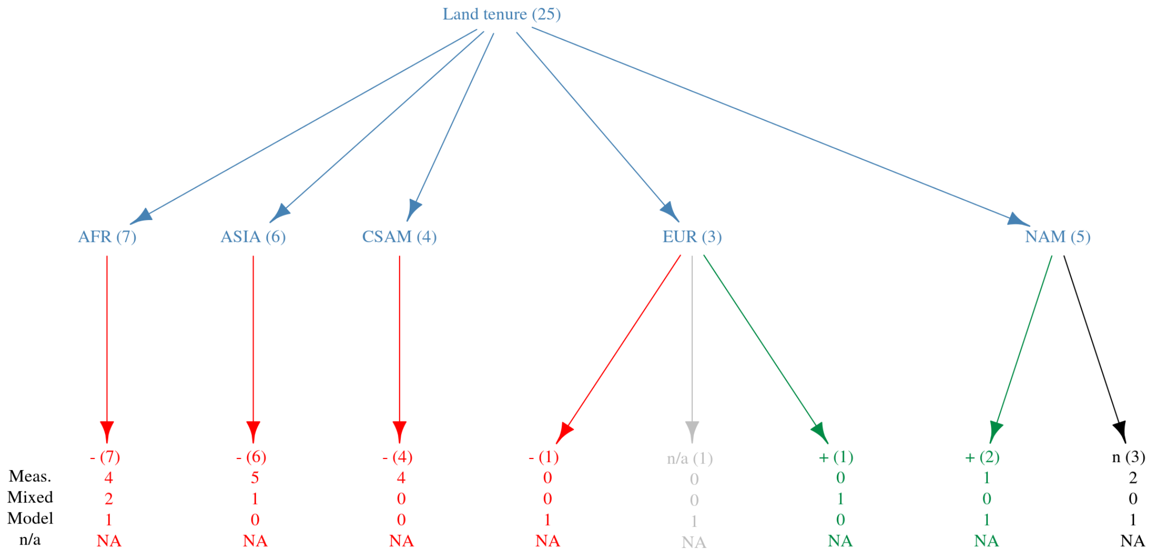

Figure S2: Regional distribution of impacts on land tenure.

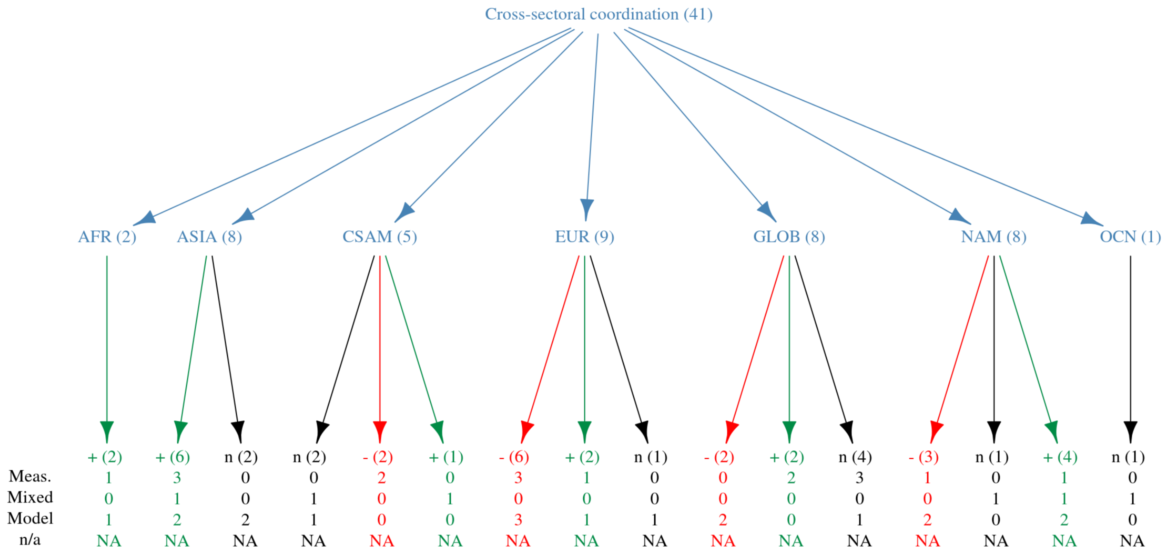

Figure S3: Regional distribution of impacts on cross-sectoral coordination.

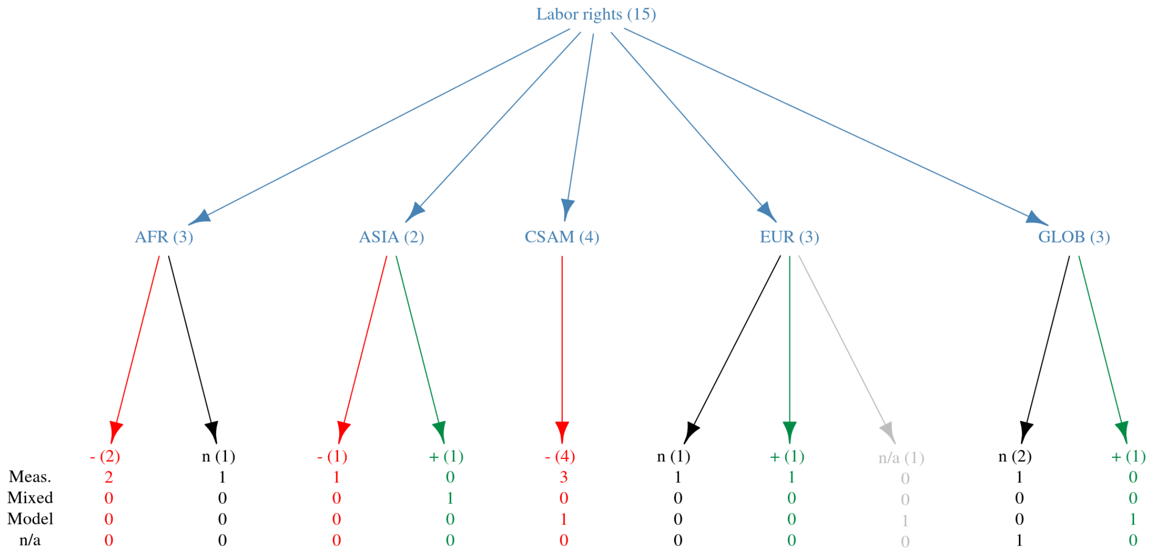

Figure S4: Regional distribution of impacts on labor rights.

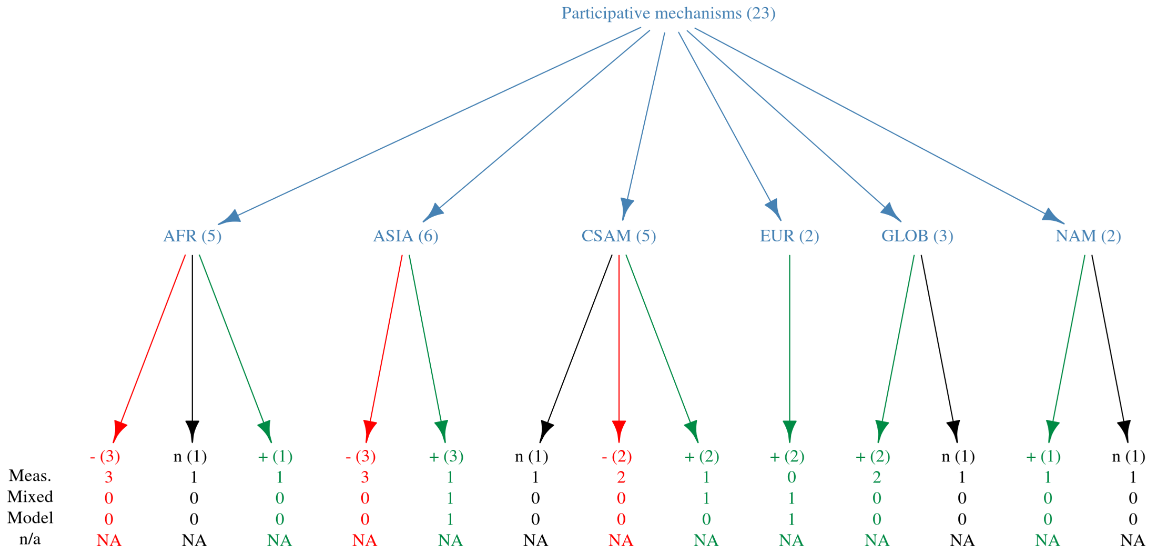

Figure S5: Regional distribution of impacts on participative mechanisms.

2 Social impacts

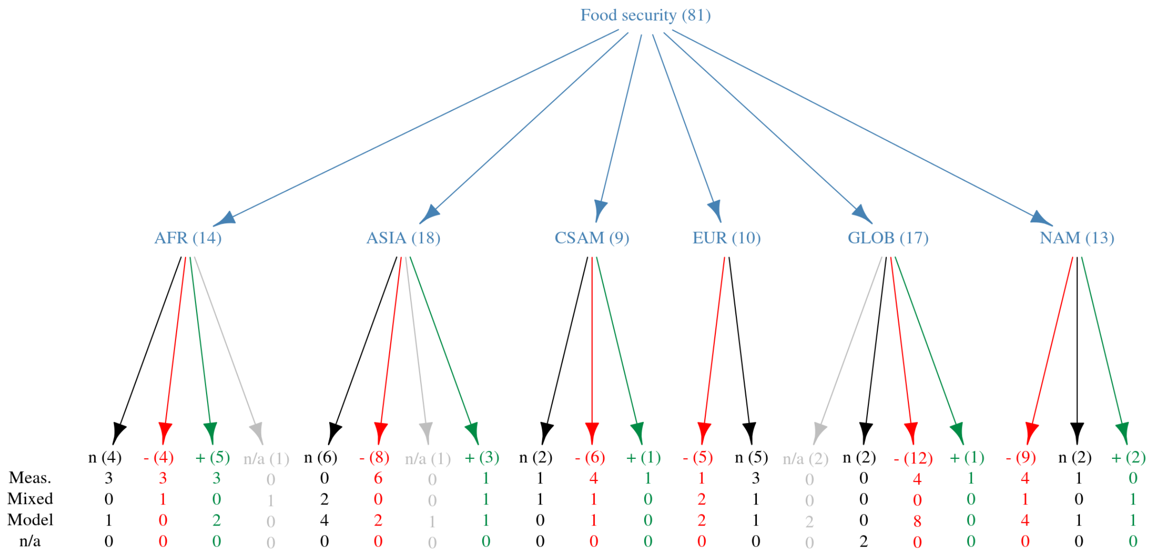

Figure S6: Regional distribution of impacts on food security.

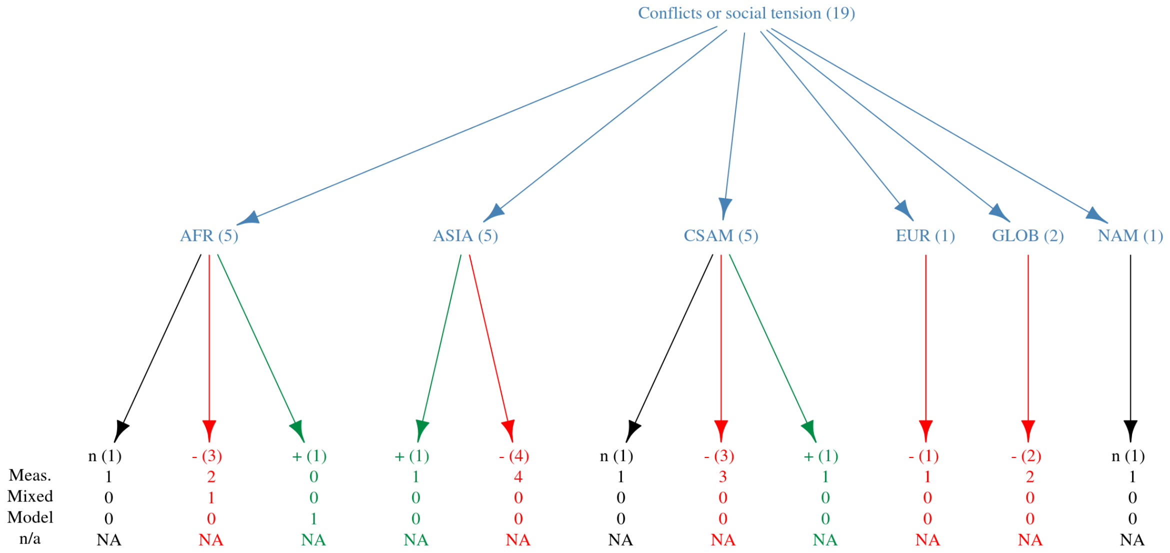

Figure S7: Regional distribution of impacts on conflicts or social tension.

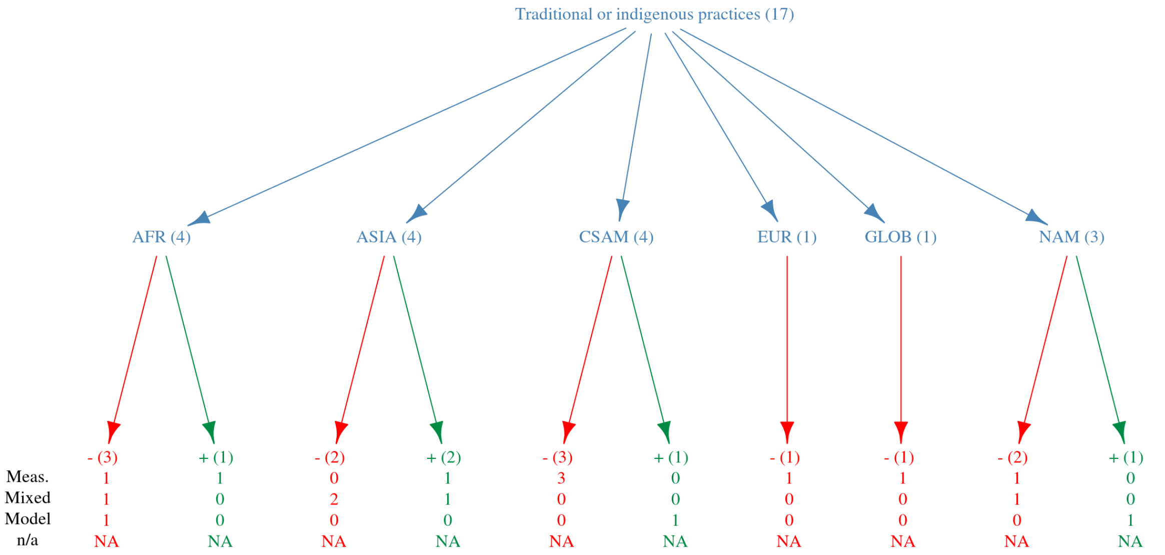

Figure S8: Regional distribution of impacts on traditional or indigenous practices.

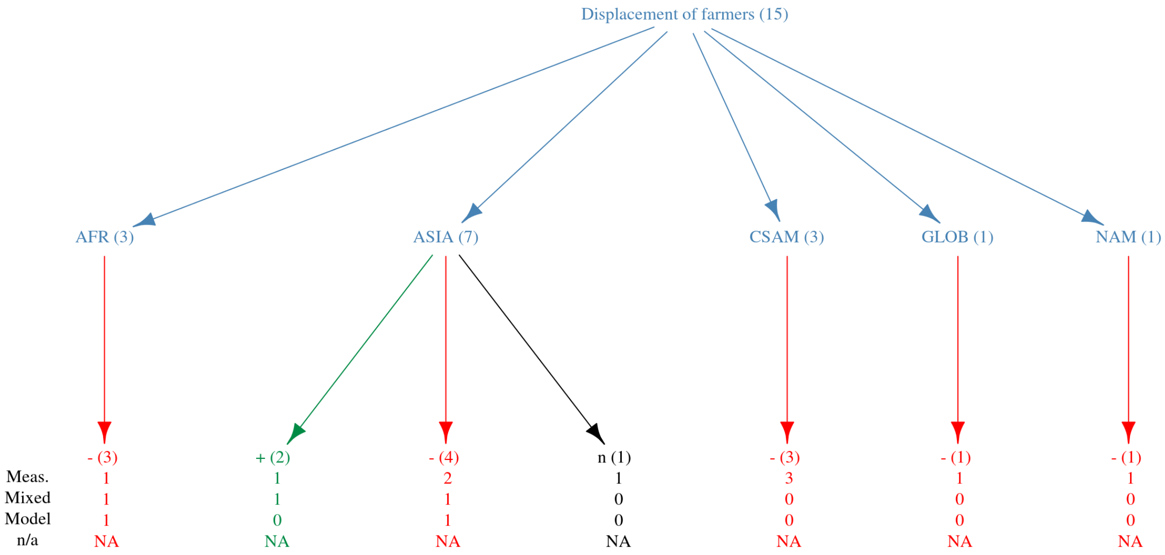

Figure S9: Regional distribution of impacts on displacement of farmers.

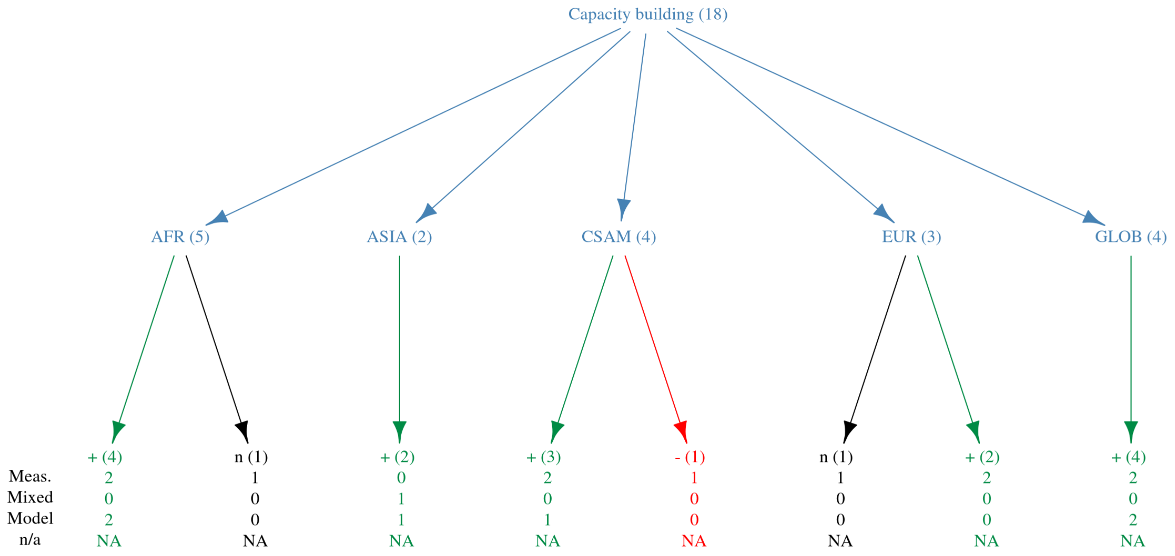

Figure S10: Regional distribution of impacts on capacity building.

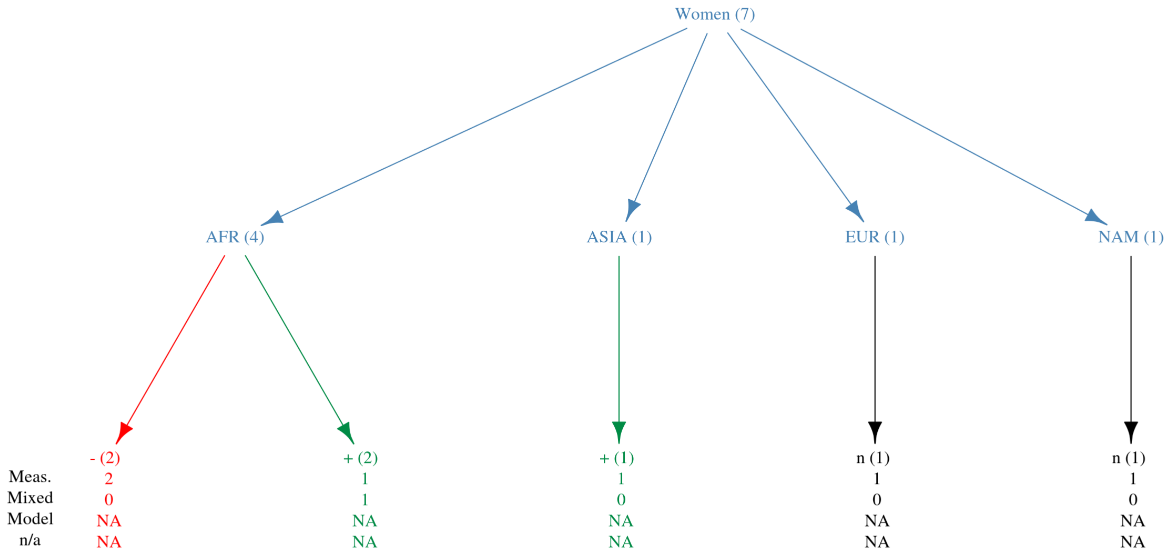

Figure S11: Regional distribution of impacts on women.

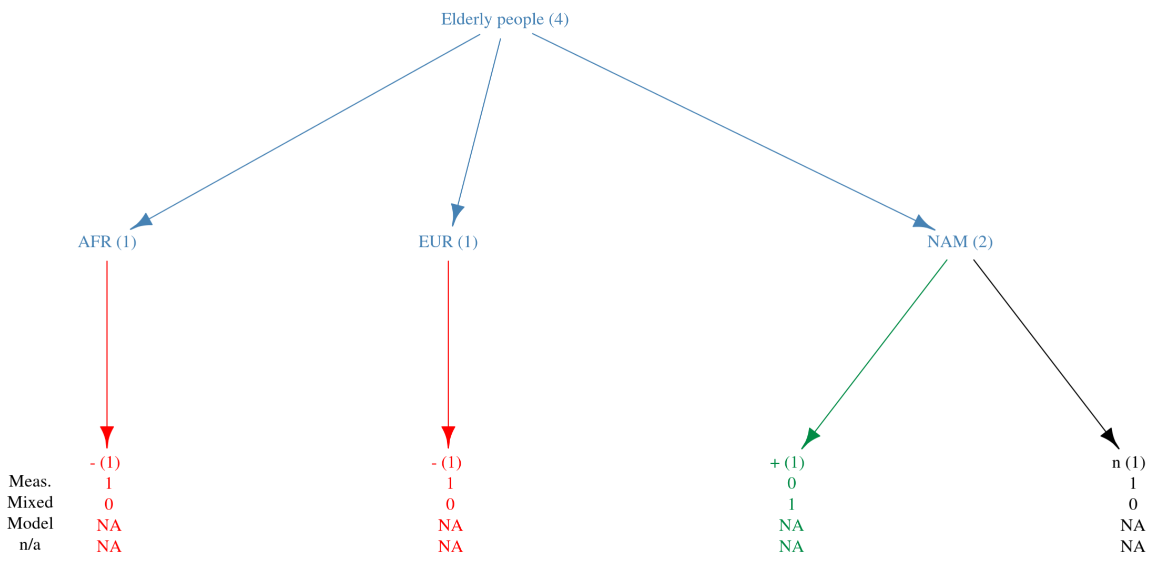

Figure S12: Regional distribution of impacts on elderly people.

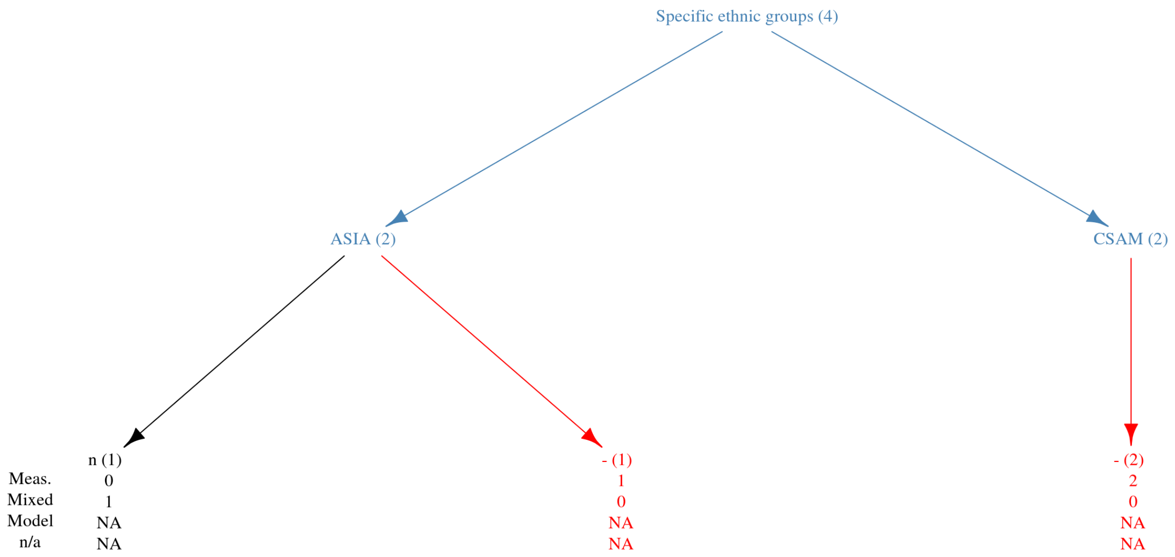

Figure S13: Regional distribution of impacts on specific ethnic groups.

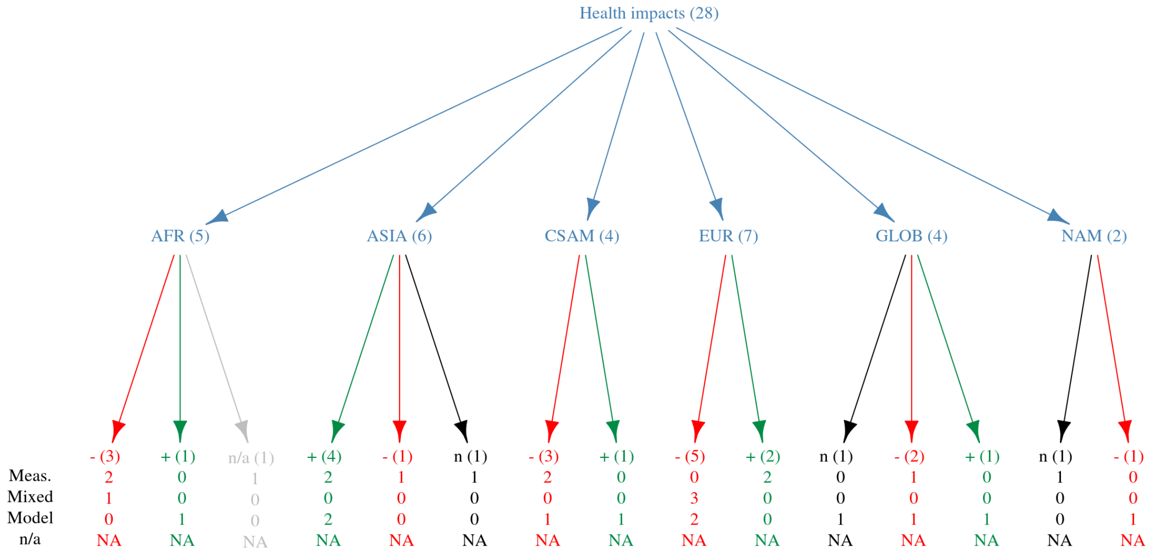

Figure S14: Regional distribution of impacts on health.

3 Environmental impacts

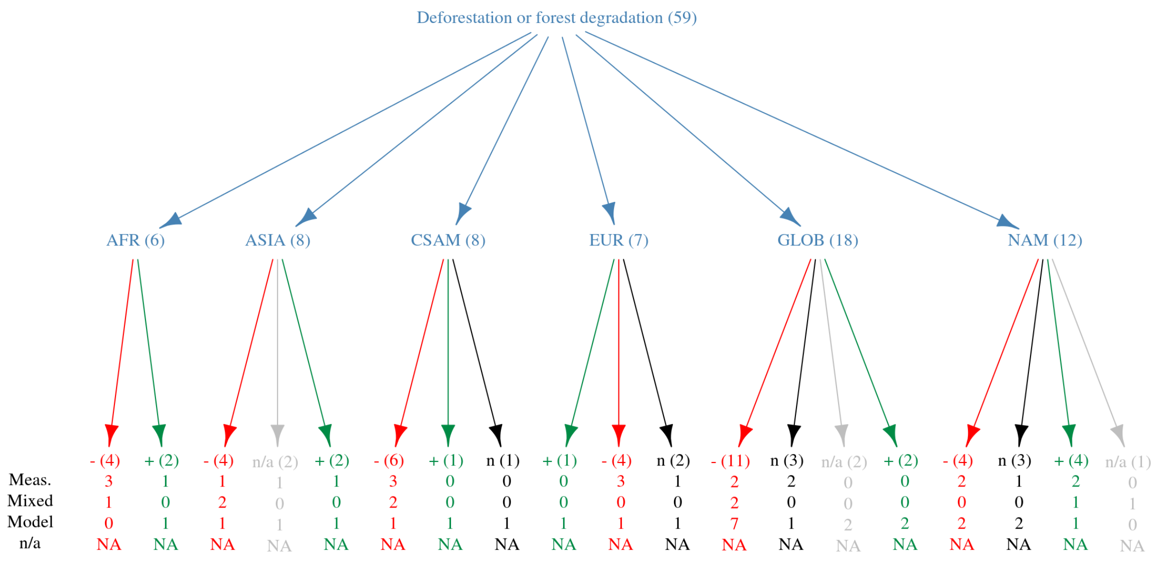

Figure S15: Regional distribution of impacts on deforestation or forest degradation.

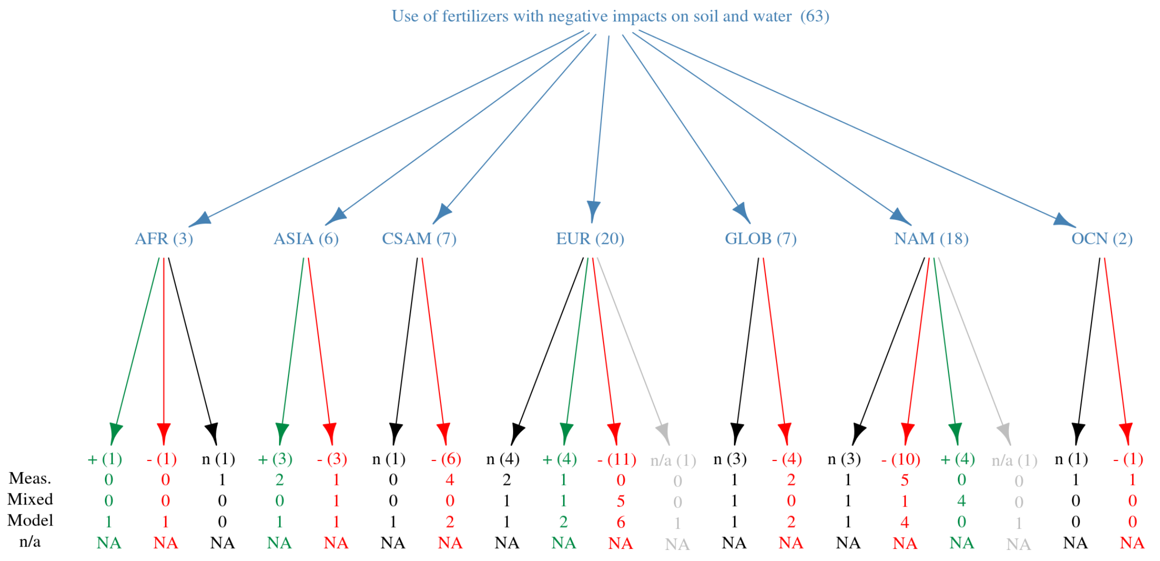

Figure S16: Regional distribution of negative impacts of fertilizers on soil and water.

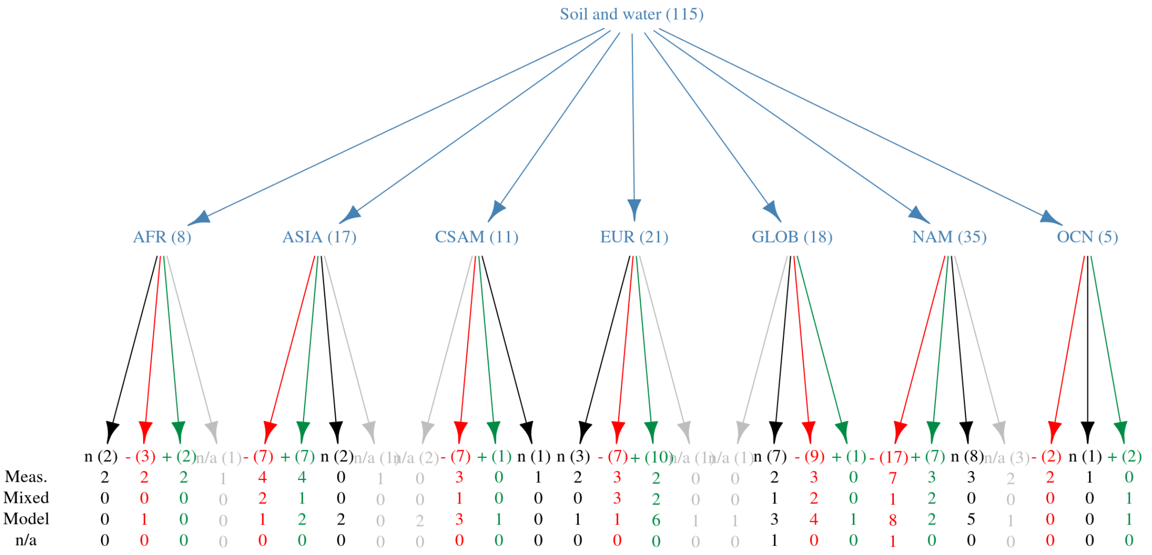

Figure S17: Regional distribution of impacts on soil and water.

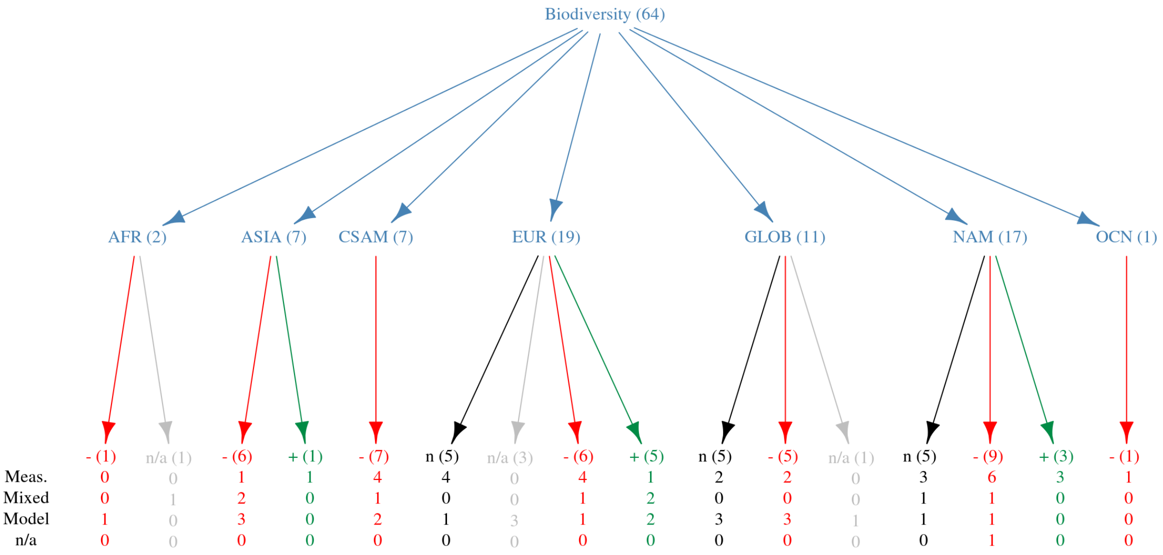

Figure S18: Regional distribution of impacts on biodiversity.

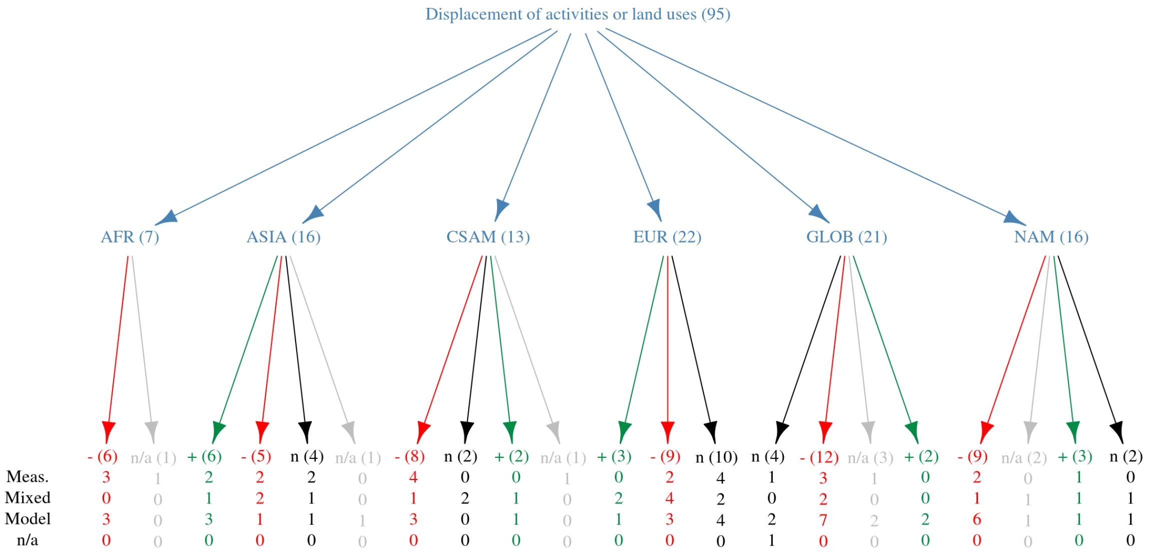

Figure S19: Regional distribution of impacts on displacement of activities or land uses.

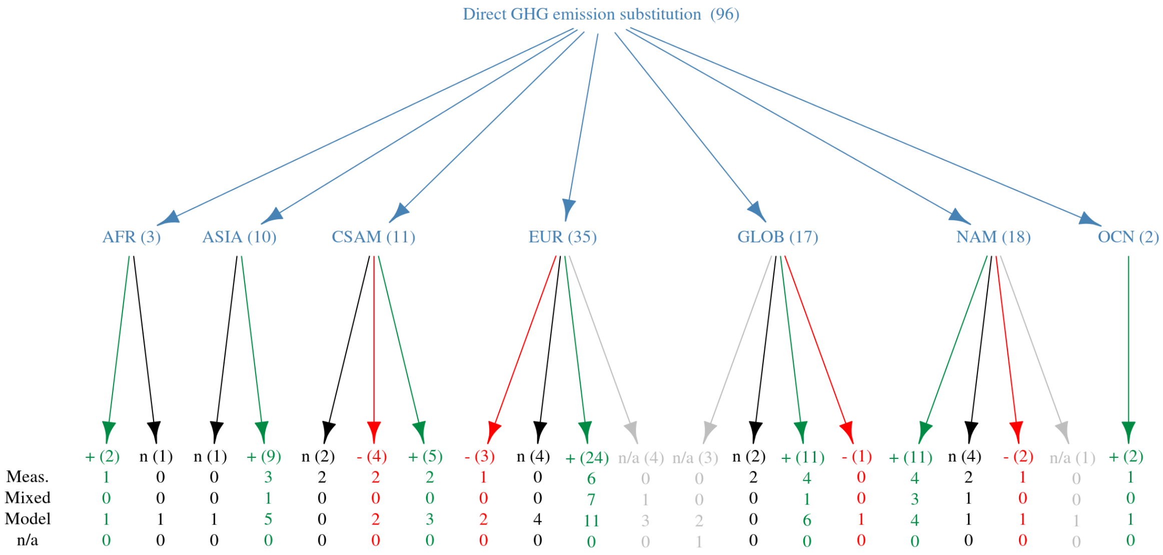

Figure S20: Regional distribution of impacts on direct GHG emission substitution.

4 Economic impacts

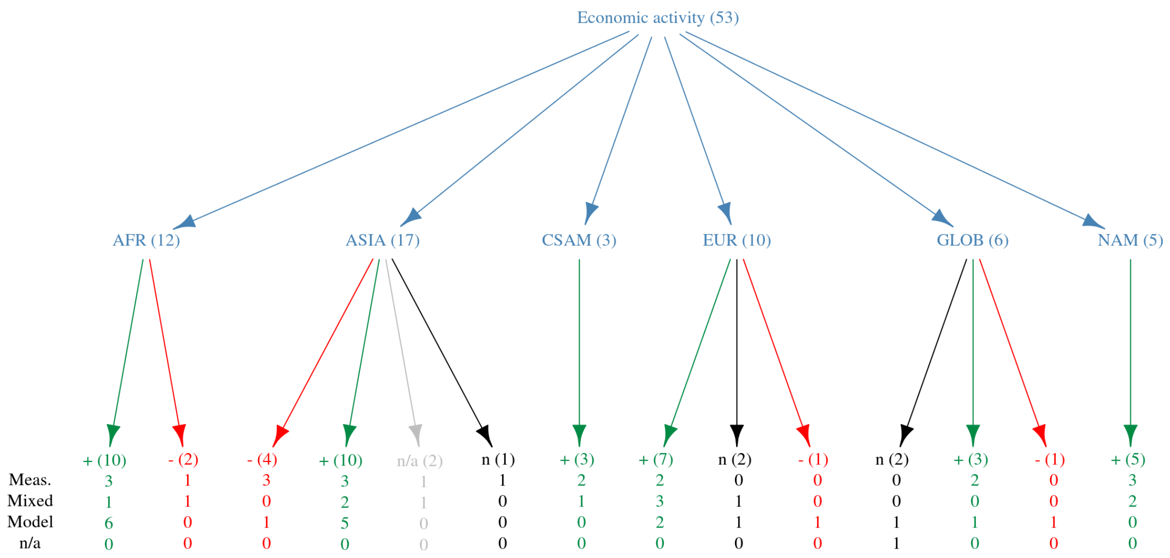

Figure S21: Regional distribution of impacts on economic activity.

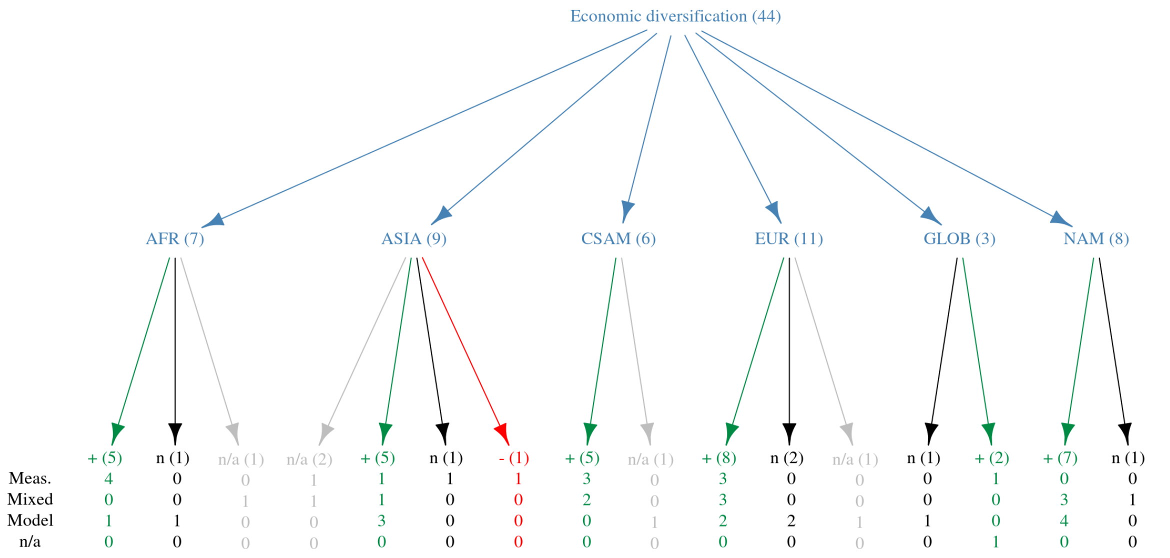

Figure S22: Regional distribution of impacts on economic diversification.

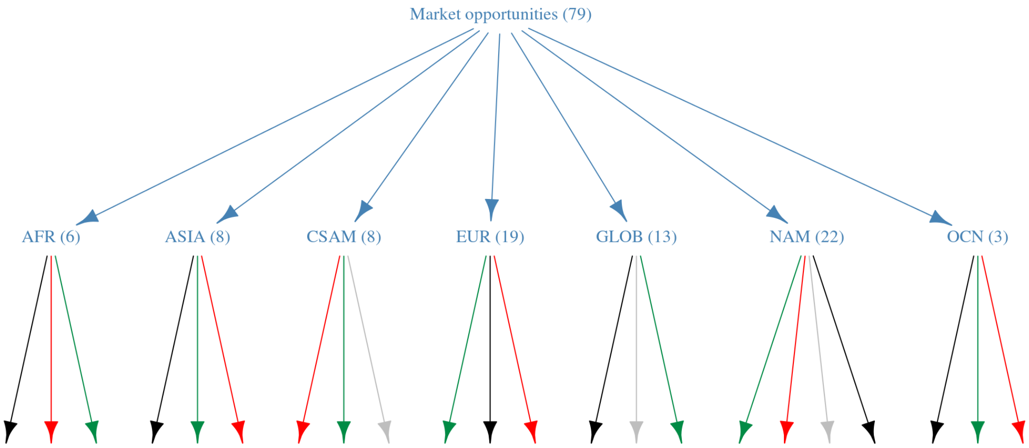

	n (2)	- (1)	+ (3)	n (2)	+ (5)	- (1)	- (2)	+ (5)	n/a (1)	+ (15)	n (3)	- (1)	n (3)	n/a (2)	+ (8)	+ (18)	- (2)	n/a (1)	n (1)	n (1)	+ (1)	- (1)
Meas.	1	1	1	0	1	0	2	3	0	6	0	0	0	1	4	3	0	0	0	1	0	1
Mixed	0	0	0	1	2	0	0	2	0	4	0	0	0	0	0	4	0	0	1	0	0	0
Model	1	0	2	1	2	1	0	0	1	5	3	1	2	1	4	11	2	1	0	0	1	0
n/a	0	0	0	0	0	0	0	0	0	0	0	0	1	0	0	0	0	0	0	0	0	0

Figure S23: Regional distribution of impacts on market opportunities.

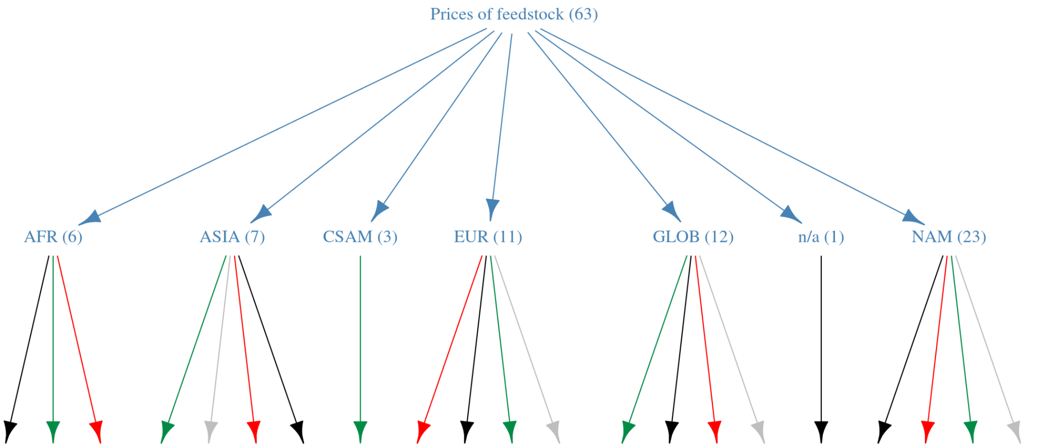

	n (3)	+ (1)	- (2)	+ (4)	n/a (1)	- (1)	n (1)	+ (3)	- (4)	n (5)	+ (1)	n/a (1)	+ (2)	n (5)	- (4)	n/a (1)	n (1)	n (7)	- (7)	+ (7)	n/a (2)
Meas.	1	0	1	1	0	0	0	2	1	0	0	0	1	0	0	0	0	1	2	2	1
Mixed	0	0	0	0	0	0	0	0	1	2	1	1	0	0	0	0	0	4	1	2	0
Model	2	1	1	3	1	1	1	1	2	3	0	0	1	4	4	1	1	2	4	3	1
n/a	0	0	0	0	0	0	0	0	0	0	0	0	0	1	0	0	0	0	0	0	0

Figure S24: Regional distribution of impacts on feedstock prices.

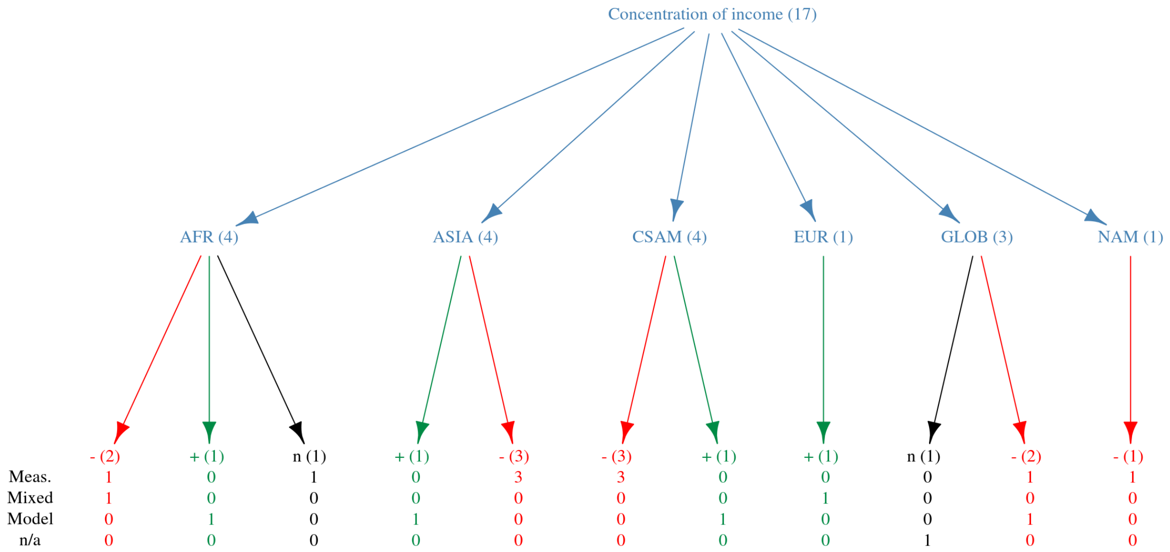

Figure S25: Regional distribution of impacts on concentration of income.

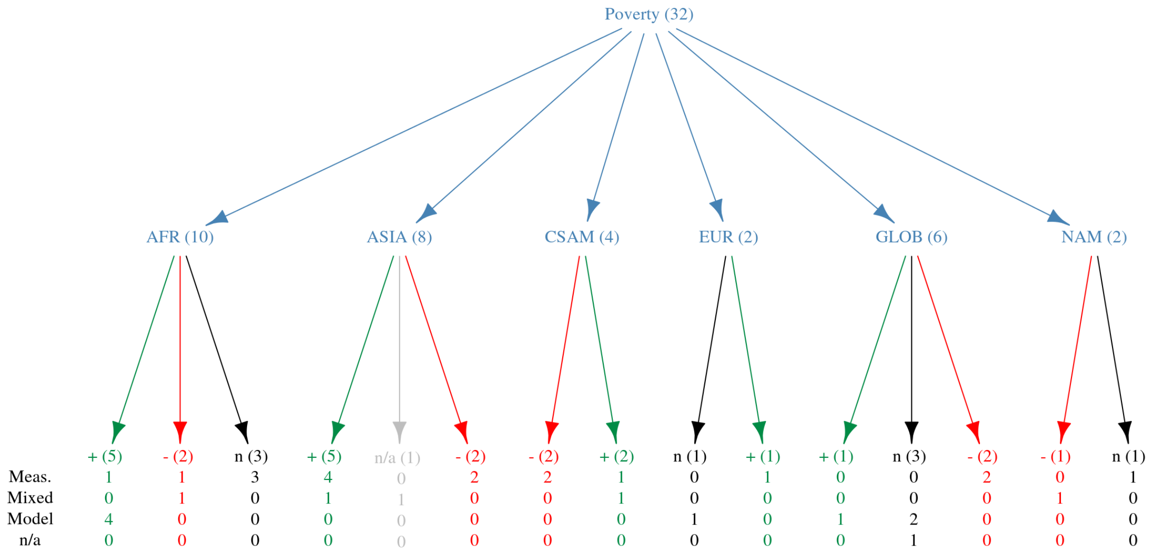

Figure S26: Regional distribution of impacts on poverty.

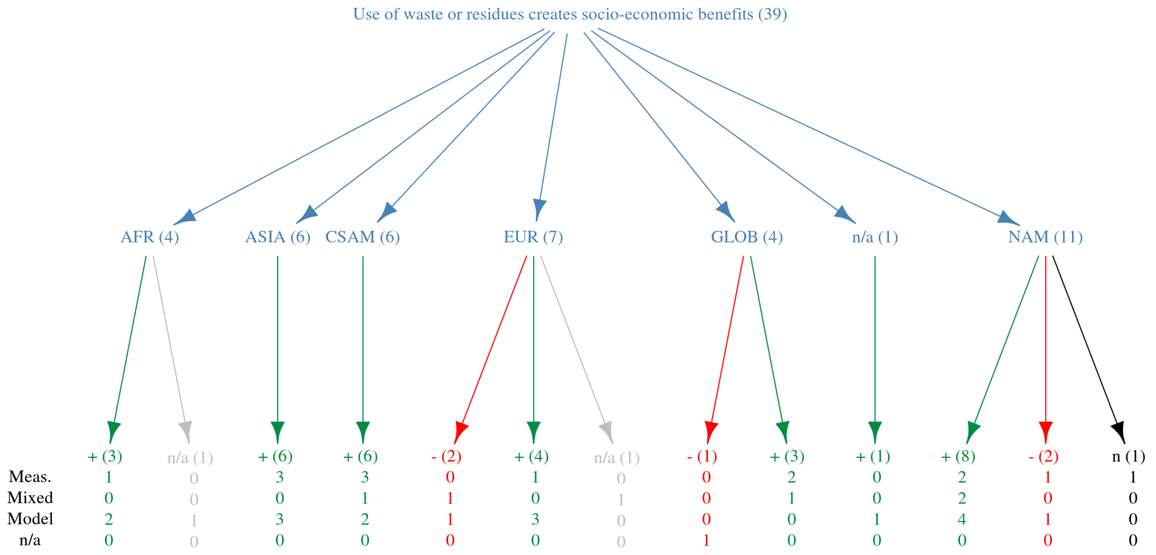

Figure S27: Regional distribution of impacts on the use of waste or residues creating socio-economic benefits.

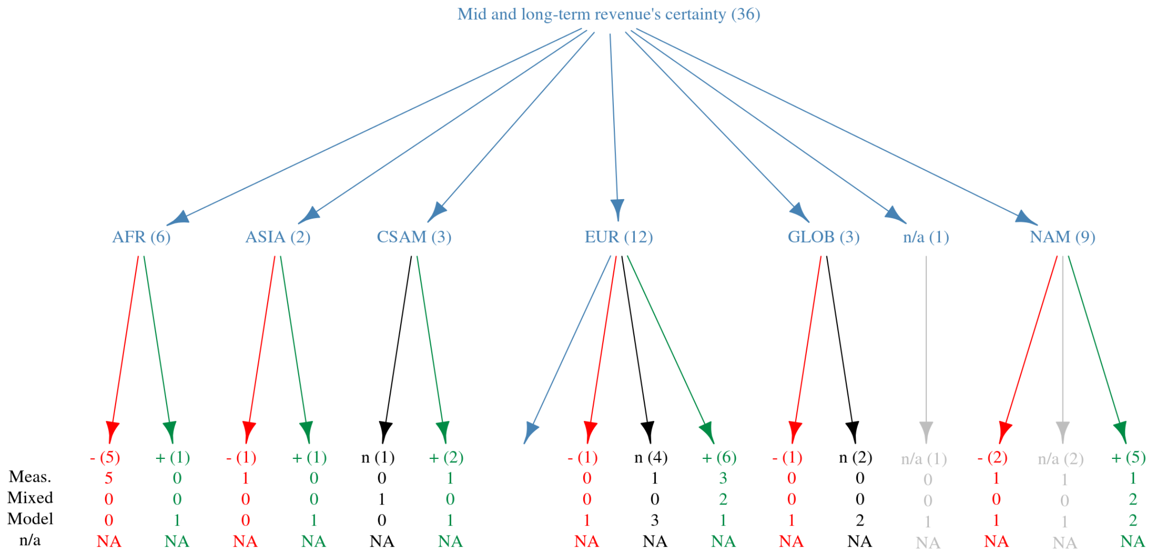

Figure S28: Regional distribution of impacts on mid and long-term revenue's certainty.

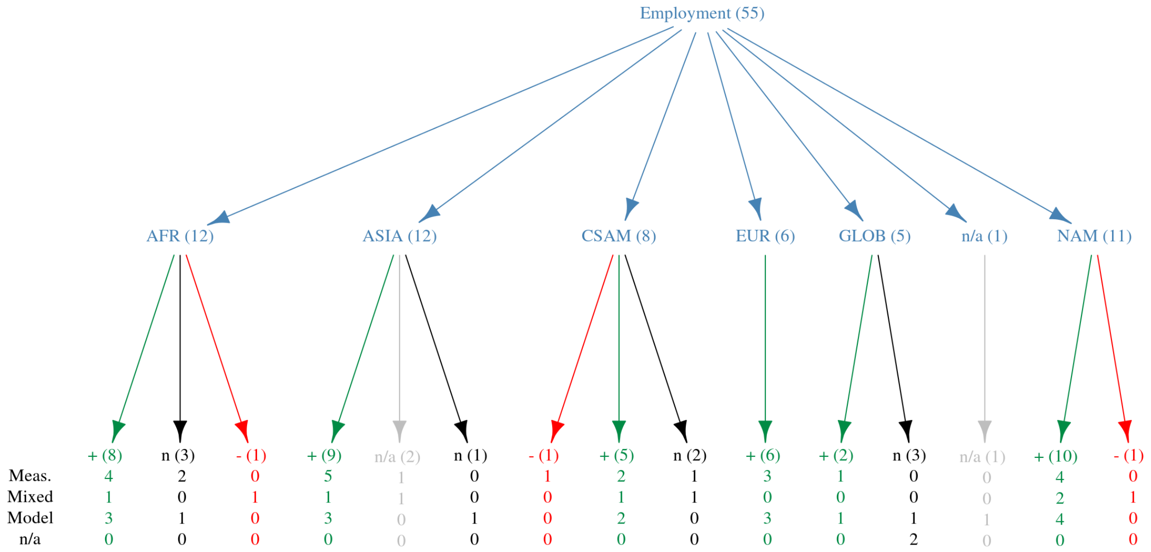

Figure S29: Regional distribution of impacts on employment.

5 Technological impacts

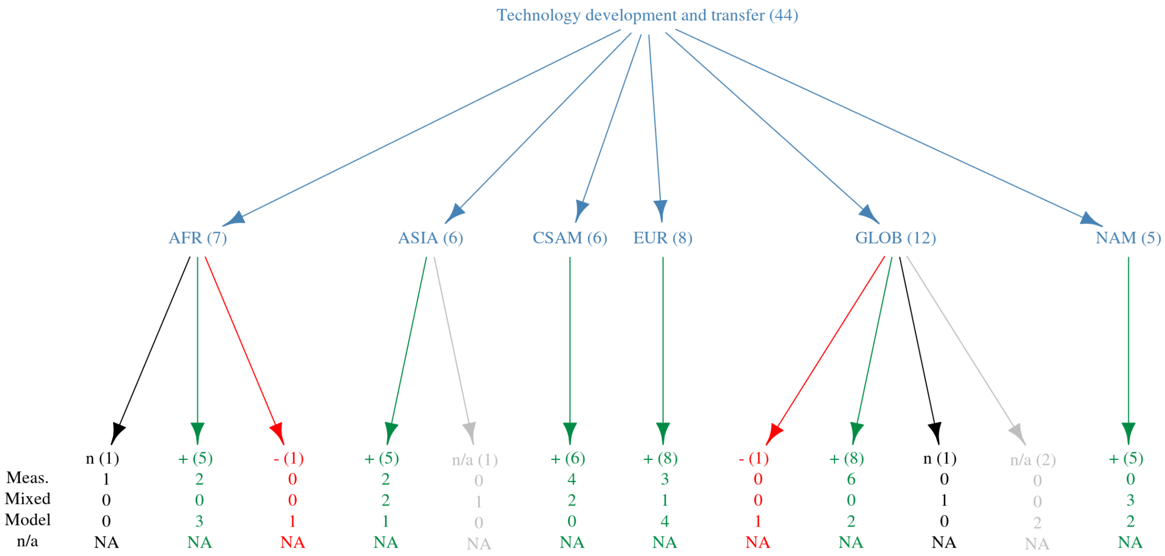

Figure S30: Regional distribution of impacts on technology development and transfer.

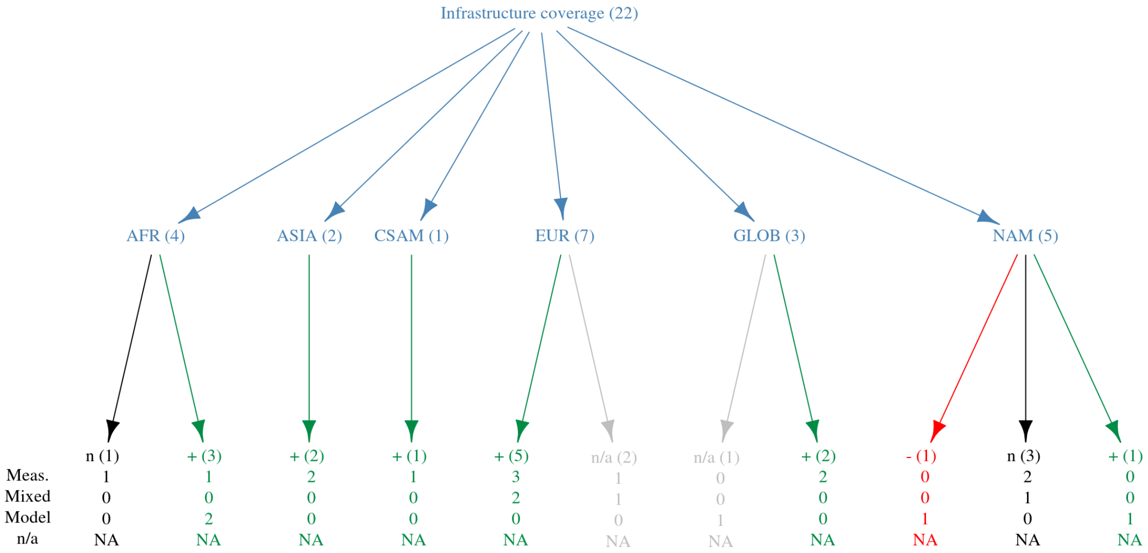

Figure S31: Regional distribution of impacts on infrastructure coverage.

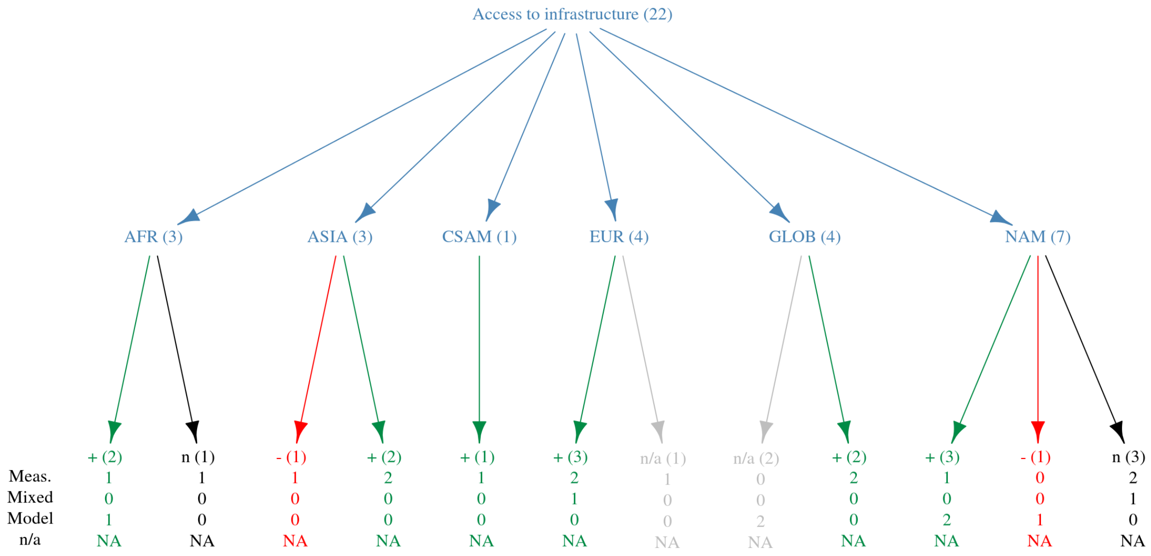

Figure S32: Regional distribution of impacts on access to infrastructure.

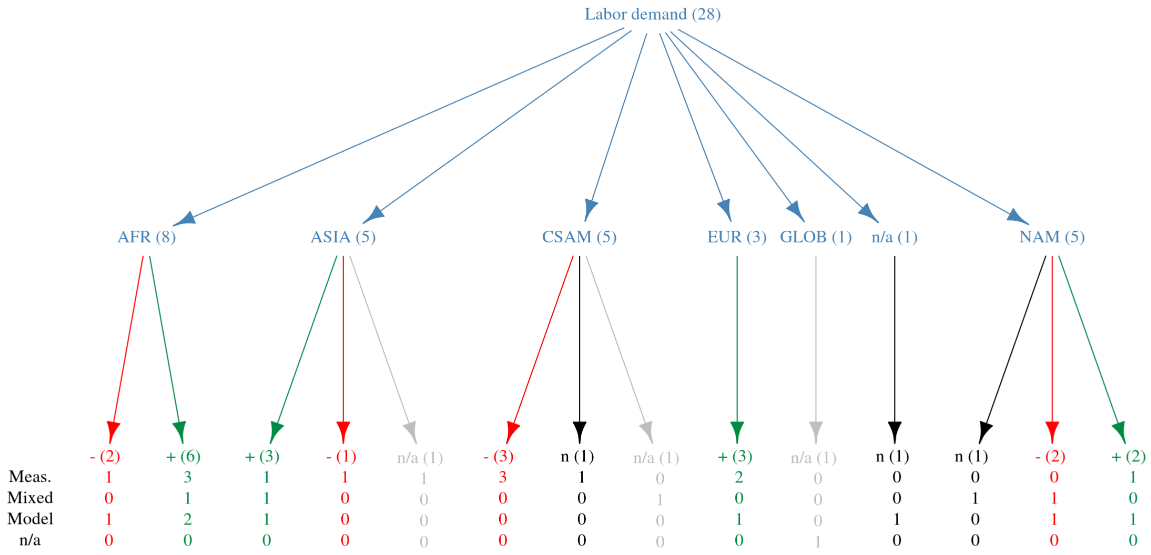

Figure S33: Regional distribution of impacts on labor demand.
