# Supplementary material for: Bioenergy production and sustainable development: science base for policymaking remains limited
Source: Glob Change Biol Bioenergy. 2016 Mar 23;9(3):541–56. doi: 10.1111/gcbb.12338 (PMC5340281; doi:10.1111/gcbb.12338)
Supplement: Supplementary file 5 [file GCBB-9-541-s005.docx]

**Data S1: Bioenergy production and sustainable development: limited science base for policy making – Protocol.**

**Data S2: Additional Results.**

**Data S3: Article selection and data extraction.**

**Data S4: Regional distribution of each impact considered in the systematic review.**
